# Spliceostatin C, a component of a microbial bioherbicide, is a potent phytotoxin that inhibits the spliceosome

**DOI:** 10.3389/fpls.2022.1019938

**Published:** 2023-01-12

**Authors:** Joanna Bajsa-Hirschel, Zhiqiang Pan, Pankaj Pandey, Ratnakar N. Asolkar, Amar G. Chittiboyina, Louis Boddy, Marylou C. Machingura, Stephen O. Duke

**Affiliations:** ^1^ Natural Products Utilization Research Unit, Agricultural Research Service, U.S. Department of Agriculture, University, MS, United States; ^2^ National Center for Natural Products Research, School of Pharmacy, University of Mississippi, University, MS, United States; ^3^ Bioceres Crop Solutions, Davis, CA, United States; ^4^ Department of Biology, Georgia Southern University, Savannah, GA, United States

**Keywords:** splicing, splicing inhibitors, spliceostatin, bioherbicide, arabidopsis, proteomics

## Abstract

Spliceostatin C (SPC) is a component of a bioherbicide isolated from the soil bacterium *Burkholderia rinojensis*. The chemical structure of SPC closely resembles spliceostatin A (SPA) which was characterized as an anticancer agent and splicing inhibitor. SPC inhibited the growth of *Arabidopsis thaliana* seedlings with an IC50 value of 2.2 µM. The seedlings exposed to SPC displayed a significant response with decreased root length and number and inhibition of gravitropism. Reverse transcriptase semi-quantitative PCR (RT-sqPCR) analyses of 19 selected genes demonstrated the active impact of SPC on the quality and quantity of transcripts that underwent intron rearrangements as well as up or down expression upon exposure to SPC. Qualitative and quantitative proteomic profiles identified 66 proteins that were significantly affected by SPC treatment. Further proteomics data analysis revealed that spliceostatin C induces hormone-related responses in Arabidopsis seedlings. In silico binding studies showed that SPC binds to a pocket between the SF3B3 and PF5A of the spliceosome.

## Introduction

Soil bacteria from the genus *Streptomyces* (gram positive) are one of the most known productive bacterial sources of recognized bioactive compounds. Starting with the discovery of actinomycin in 1945 (Jones et al., 1945), thereafter, multiple bioactive secondary metabolites with antibiotics (about 80% of discovered antibiotics), anticancer, anti-inflammatory or antifungal properties have been identified (Watve et al., 2001; Traxler and Kolter, 2015). Recently, the *Burkholderia* genus (gram-negative) has received significant attention from industrial biotechnology (Eberl and Vandamme, 2016; Thapa and Grove, 2019). *Burkholderia* species thrive in many environments, including soil, plants, insects, and mammals, establishing either antagonistic or mutualistic interactions with their hosts. The ability of *Burkholderia* spp. to adapt to diverse environments is undoubtedly a result of large genome sizes that include predicted multiple gene clusters involved in the biosynthesis of secondary metabolites (Liu and Cheng, 2014). Intriguingly, some *Streptomyces* and *Burkholderia* strains biosynthesize a variety of secondary metabolites with similar splicing inhibitor properties. The biosynthesis of complex secondary products involves significant energy utilization by the bacteria, and such efforts must translate to substantial survival benefits. In many cases, natural products are highly specific, and their mode of action is sophisticated. In this context, splicing inhibitors are directed specifically against eukaryotic organisms present in the same environment since most bacterial genomes do not contain introns, and only rare examples show their presence, but not with typical eukaryotic introns and spliceosome machinery (Lamolle and Musto, 2018).

The structurally complex splicing inhibitors, including pladienolides (*S. platensis*), herboxidiene (HERB) (*S. chromofuscus*), spliceostatins (*B. rijonensis*) and thalianstatins (*B. thaliandensis*) share significant structural similarities (**Supplementary Figure 1**) such as the diene moiety located in the middle of the structure (Liu et al., 2013b; He et al., 2014; Pokhrel et al., 2015). This diene moiety is apparently the key feature enabling the positioning and interaction with certain spliceosome proteins. Another common characteristic of pladienolides and herboxidiene is an aliphatic arm, while tetrahydropyran is found in structures of herboxidiene and spliceostatins (Pokhrel et al., 2015). In addition to the compounds mentioned above, numerous derivatives of these natural products and analogs have been synthesized and found to be splicing inhibitors (Effenberger et al., 2014; Lagisetti et al., 2014). There is interest in these chemicals due to their high antitumor activity against MCF-7 human mammary adenocarcinoma cells and their ability to reduce cholesterol levels (Nakajima et al., 1996; Kaida et al., 2007).

In 2007, Kaida and coauthors discovered that spliceostatin A (SPA) inhibited the spliceosome splicing mechanism (Kaida et al., 2007). In the study, they used a biotinylated version of SPA to identify proteins interacting with this chemical and detected the presence of the U2 SF3b spliceosome complex proteins SAP155, SAP145, SAP130 and SAP49. Splicing is a very dynamic and intricate maturation of a precursor mRNA (pre-mRNA); i.e., an intron-removing process occurring in the nucleus. The entire process is facilitated by small nuclear RNAs (snRNA) that, together with specific proteins, create ribonucleoprotein (RNP) complexes (Wang and Brendel, 2004). The Arabidopsis genome contains 74 such snRNAs and about 430 other genes encoding known and putative proteins of the spliceosome apparatus (Koncz et al., 2012). Simplifying, two Sn2-type transesterification reactions are essential to remove an intron, but its separation is orchestrated by an exceedingly large number of proteins and snRNAs. The majority of genes in plant and animal genomes contain introns (Mollet et al., 2010).

Splicing is an important form of transcriptional regulation that was underestimated in the past. Frequently, pre-mRNA undergoes non-canonical alterations like intron retention, exon skipping, alternative 5’ or 3’ splicing sites, etc. that lead to generating proteins with alternative functions/characteristics or simply non-functional proteins from a single gene. This phenomenon is known as alternative splicing (AS) that enables genomes to extend beyond the constraints of their length (nucleotides). Regulation of AS in plants is highly dependent on tissue specificity and/or external conditions (Klepikova et al., 2016). Additionally, the frequency of utilized particular types of AS depends on the type of organism and/or species (Chaudhary et al., 2019). AS-dependent regulation of gene expression in response to fluctuations of environmental conditions plays a significant role in defining an assortment of adaptations that enhance plant survival. In plants, AS is an element of complex, multilevel regulation of circadian cycles, flowering, and responses to biotic and abiotic stress (Balasubramanian et al., 2006; Seo et al., 2012; Liu et al., 2013a). AS appears to be more involved in the stress responses of plants than of animals (Martin et al., 2021). Abscisic acid is the primary plant hormone responsible for the modulation of plant response to stress and this process is regulated by posttranslational modifications and AS (Laloum et al., 2018).

**Figure 1:**
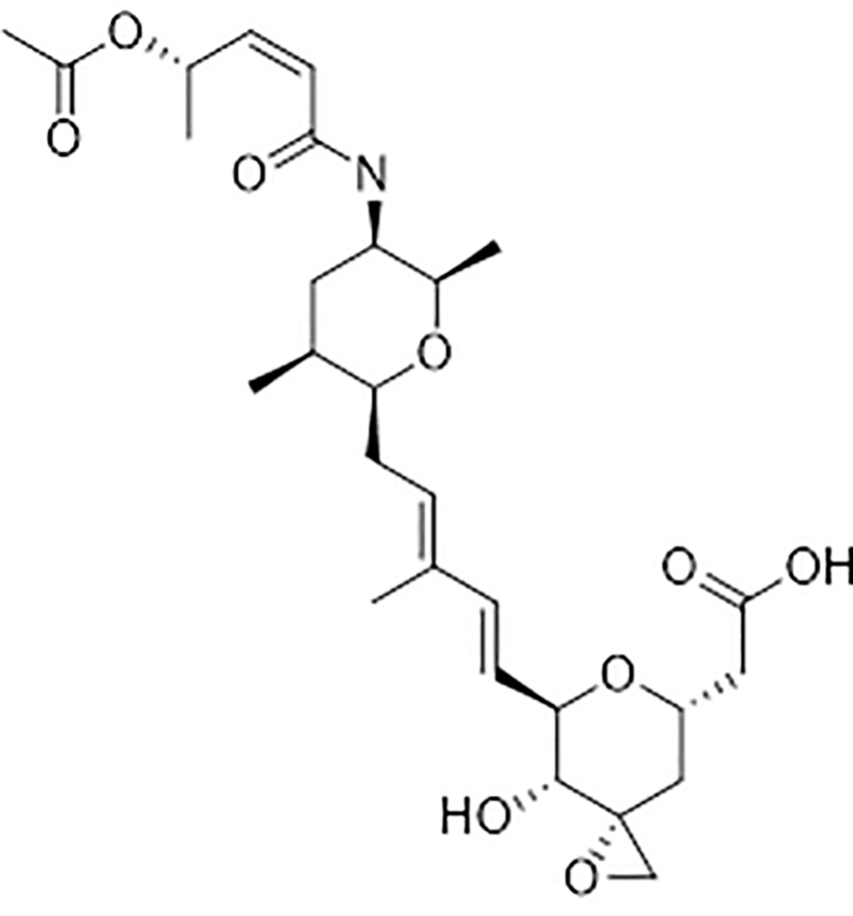
Chemical structure of Splicing inhibitor: spliceostatin C.

Because of the widespread evolution of resistance to herbicides, there is a strong demand for herbicides with new modes of action (Duke and Dayan, 2021). Spliceostatin C (SPC) (**Figure 1**) is one of the bioactive components of a bioherbicide produced from the soil bacterium *Burkholderia rinojensis* (Marrone, 2019). Another significant bioactive component of this bioherbicide is romidepsin, an inhibitor of histone deacetylases (Owens et al., 2020). The main objective of this study was to investigate the response of *Arabidopsis thaliana* seedlings to SPC and to characterize its herbicidal mode of action. SPC was highly phytotoxic at low doses. Here, we confirmed that this chemical disrupts the splicing process, modifying transcript lengths and their expression levels, as well as altering resulting proteins.

## Materials and methods

### Plant material and growth conditions

Arabidopsis seeds of ecotype Columbia (Col-0) were purchased from Lehle Seeds Company (Round Rock, TX). Plants were grown in a growth chamber using a 16 h at 24°C (day) and 8 h at 22°C (night) regime at a light intensity of 125 µmol m^-2^ s^-1^ PAR.

### Chemicals

The splicing inhibitor herboxidiene (HERB) was purchased from Focus Biomolecules, and spliceostatin C (SPC) was provided by Bioceres Crop Solutions. The quantitative determination of the hormone abscisic acid (ABA) was done with a Phytodetek^®^ ABA Test Kit (Agdia, Inc., Elkhart, IN).

### Dose-response bioassay

1 ml of autoclaved half-strength MS (Sigma-Aldrich, St. Louis, MO) medium (pH 5.7) with 0.8% phytagel (PlantMedia, St. Gardena, CA) was transferred into each well of 24 well plate (Corning, Inc., Corning, NY). After the medium solidified, five sterile Arabidopsis seeds were sown into each well, and then plates were moved to a cold room and incubated at 4°C for 4 days. The stratified seeds were transferred to a growth chamber (16 h at 24°C - light and 8 h at 22°C - dark) to germinate. Seven-day-old seedlings were soaked in SPC or HERB at the concentrations of 0 (solution control), 0.01, 0.33, 1, 3.3, 10, 33, and 100 µM for 5 min. Each concentration was performed in three wells. Treated plants were moved back into the growth chamber and grown for 7 days. The leaf surface area was measured daily with a Scanalyzer (LemnaTec, Aachen, Germany). All inhibitors and control treatments were performed in triplicate.

### Root phenotyping

Surface sterilized and stratified Arabidopsis seeds were plated in the square petri dish containing half-strength MS and 0.8% phytagel (25 seeds per plate). After seven days of growth in the conditions described above, Arabidopsis seedlings were treated in SPC (15.2 µM) or HERB (1.72 µM), IC_80_ concentrations. After 5 min, the solution was removed, and plates were transferred to the growth chamber for seven days. The root length was measured with ImageJ software.

### Gravitropism assay – root tip reorientation

The protocol is a modified version described by Roy et al. (Roy and Bassham, 2015). The growth conditions and treatment were similar to those described above. After the treatment, plates were transferred to a growth chamber and rotated by 90°. Photos were taken 6 and 24 h after exposure to SPC and HERB. The root tip reorientation and the angles of root tips were evaluated with ImageJ software.

### Stomatal aperture measurement

The analysis of stomatal aperture size was performed as described by Bright et al. (Bright et al., 2006). Whole leaves detached from three week old Arabidopsis plants were incubated in MES/KCl buffer (5 mM KCl, 10 mM MES, 50 μM CaCl_2_, pH 6.15) for 3 h. Each sample contained two leaves and 15 stomata was measured per leaf. All treatments were performed in triplicate. The IC_80_ concentration of splicing inhibitors was added and incubated for 30 min. ABA solution was then added to a final concentration of 20 μM. Four hours after exposure, photos were captured using a light microscope (BX60, Olympus) and the stomatal aperture index (SAI) was calculated by stomatal width/length ratio using ImageJ software.

### Treatment of Arabidopsis seedlings for RT-PCR and proteomic analyses

About one hundred and fifty Arabidopsis seeds were planted in a petri dish containing half-strength MS and 0.8% phytagel and germinated in a plant growth chamber. Seven-day-old seedlings were soaked with spliceostatin C solution (IC_50_ - 2.2 µM or IC_80_ - 15.2 µM) or herboxidiene (IC_50_ - 0.37 µM or IC_80_ - 1.72 µM) containing 0.015% DMSO and 0.05% Tween 20 for 5 min. The control solution contains DMSO and Tween 20 only. Seedlings were harvested 6 and 24 h after removing the treatment solution. Each treatment was replicated three times.

### Reverse Transcriptase semi-quantitative PCR (RT-sqPCR) analyses

Total RNA was isolated using the RNeasy Plant Mini Kit (Qiagen, Valencia, CA) according to the manufacturer’s instructions. The RNA samples were treated with DNase I (Qiagen) to remove residual DNA. The integrity of purified total RNA was confirmed by 1% agarose gel electrophoresis with SYBR GREEN staining. The concentration and purity of total RNA were determined spectrophotometrically (NanoDrop One, Thermo Fisher Scientific, Waltham, MA) at wavelengths of 260 and 280 nm. First-strand cDNA was synthesized from 500 ng of total RNA in 20 µl of reaction using the Superscript IV First Strand cDNA Synthesis Reaction kit (Invitrogen, Waltham, MA). Primer pairs for the semi-quantitative PCR covering either the full-length sequences or their fragments were designed using Geneious 10.0.5 software (**Supplementary Table 1**). PCR reactions were carried out using Platinum™ SuperFi™ PCR Master Mix (Invitrogen). The PCR products analysis were analyzed on 1.5% agarose gel with SYBR GREEN staining (Thermo Fisher Scientific).

To verify the PCR products, amplicons from RT-sqPCR were ligated to pMiniT vector (New England BioLabs, Ipswich, MA). The ligated vectors were then transformed into TOP10 *E. coli* cells using the One Shot Chemically Competent kit (Invitrogen). Plasmid DNAs were isolated and subjected to sequencing analyses. DNA sequencing was conducted by the USDA-ARS-SEA Genomics and Bioinformatics Research Unit.

### Quantitative Reverse Transcription PCR (RT−qPCR) reaction

Total RNAs were isolated from flash frozen Arabidopsis tissues using an RNeasy plant mini kit (Qiagen) according to the manufacturer’s instructions. RNAs were then treated with RNase-free DNase I kit to remove residual DNA contamination and repurified with RNeasy MinElute Cleanup Kit (Qiagen) according to the manufacturer’s procedures. RNA recovery and purity were determined spectrophotometrically using a NanoDrop One spectrophotometer (Thermo Scientific). The quality and quantity of prepared total RNAs and evaluation of qPCR experiments were accessed according to the MIQE Guidelines (Bustin et al., 2009; Bustin et al., 2010). First-strand cDNA was synthesized using iScript Advanced cDNA Synthesis Kit for RT-qPCR (Bio-Rad) using 1 µg of total RNA as template according to the manufacturer’s instruction. Primers with a melting temperature of 60°C were designed using Primer-BLAST (https://blast.ncbi.nlm.nih.gov/) with default settings (**Supplementary Table 2**). PCR was performed in triplicate using CFX96 Touch™ Real-Time PCR Detection System (Bio-Rad). Briefly, the qPCR reactions were conducted in a final volume of 20 µL containing 5 µL of diluted first strand cDNA, 5 pmol of each forward and reverse primer, 10 µL iTaq Universal SYBR Green Supermix (Bio-Rad) with conditions of 95°C for 30 s, 40 cycles of 95°C for 5 s, 60°C for 30 s, and then increasing the temperature by 0.5°C every 5 s to access the product Melt Curve according to the recommendations of the manufacturer. To obtain the primer efficiency curves, qPCRs were conducted using a 10-fold serial dilution of cDNA samples, ranging from 0.0001 ng to 100 ng (the equivalent of 0.0001 ng to 100 ng total RNA). Primer efficiency and slope were then calculated using Bio-Rad CFX Manager software (version 3.1). A range from 91.6% to 99.4% for primer efficiency and -3.299 to -3.511 for slope were obtained for all primers used in the experiments. All R2 values were greater than 0.995. For each sample in qPCR experiment, 5 µL of diluted first strand cDNA (the equivalent of 10 ng of total RNA) was used in the reaction. All values were normalized to the expression values of two reference genes (UBQ10 and UBC) according to Czechowski et al. (2005).

### Homology Modeling and Docking Study

The complete sequences of SF3B1 (UniProtKB: Q9FMF9) and PHF5A (UniProtKB: P0DI19) were obtained from the UniProt website (https://www.uniprot.org). The primary sequences of SF3B1-PHF5A subunits of *Arabidopsis thaliana* and template (PDB ID: 6EN4) were aligned using the Clustal W algorithm. Following alignment, the subunits of human SF3B1-PHF5A (6EN4) showed 84% sequence identity and 92% similarity with SF3B1-PHF5A subunits of *Arabidopsis thaliana*. A total of 10 homology models were built using the knowledge-based method implemented in the Schrödinger Suite 2020 (Schrödinger Release 2020-4: Maestro, Schrödinger, LLC, New York, NY, 2020). The co-crystalized ligand from the 6EN4 template, pladienolide B, was retained during model generation. The Prime (Jacobson et al., 2004) module implemented in the Schrödinger suite was used to refine the loops region of generated models.

The Ramachandran plot analysis was used to assess the quality of the generated models (Ramachandran et al., 1963). All the amino acids except Ala1047 were present in the favored/allowed regions in the Ramachandran plot, which facilitated the use of the best homology model for the docking study (**Supplementary Figures 2A, B**). The three-dimensional (3D) homology model of SF3B1-PHF5A subunits of *Arabidopsis thaliana* was subsequently prepared by adding hydrogens, adjusting bond orders, and ionizing at physiological pH of 7.4 using the “Protein Preparation Wizard” (Sastry et al., 2013) module in the 2020-4 Schrödinger suite. The 2D structures of spliceostatin C and pladienolide B were sketched in Maestro (Schrödinger Release 2020‐4: Maestro, Schrödinger, LLC, New York, NY, 2020) and 3D energy-minimized using the “LigPrep” (Schrödinger Release 2020‐4: LigPrep, Schrödinger, LLC, New York, NY, 2020) module of the Schrödinger suite through the Optimized Potential for Liquid Simulations 3e (OPLS3e) force field (Harder et al., 2016). Finally, the docking of SPC and pladienolide B into the best 3D homology model of SF3B1-PHF5A subunits of *Arabidopsis thaliana* was performed using the extra-precision (XP) (Friesner et al., 2006) method implemented in the Glide (Friesner et al., 2004) module of the Schrödinger software. The grid for the 3D homology model of SF3B1-PHF5A was prepared using the centroid of the co-crystallized ligand (Pladienolide B) in the homology model.

Additionally, the van der Waals radius-scaling factor and partial charge cutoff remained at 1 and 0.25, respectively. No additional constraints were applied during the preparation of the grid and docking process. Furthermore, the binding free energies (Prime MM-GBSA free energy (Jacobson et al., 2002) of the docked structures were calculated using the Prime module of the Schrödinger software, allowing the flexibility of side-chains within 5Å of the ligand. To further understand the impact of the interaction between Tyr36 (Y36) and SPC, the wild-type Y36 of the homology model of SF3B1-PHF5A was computationally mutated into Y36L, Y36W, Y36A, Y36C, Y36R, and Y36E. These mutated models were subsequently energy-minimized for further docking and binding free-energy calculations with SPC.

### Protein isolation and 2-D DIGE protein profiling

One-week-old Arabidopsis seedlings were treated with SPC as described above. The proteome analysis was conducted by Applied Biomics, Inc. (Hayward, CA). Briefly, Arabidopsis seedlings were ground to a powder in liquid nitrogen, and 2-D cell lysis buffer (30 mM Tris-HCl, pH 8.8, containing 7 M urea, 2 M thiourea and 4% CHAPS) was added to each sample, and then sonicated on ice, followed by shaking at room temperature for 30 min. The samples were then centrifuged at 25,000 x g at 4°C for 30 min and the supernatant (protein lysate) was collected. Protein quantitation was carried out using a Bradford protein assay kit (Bio-Rad, Hercules, CA). The protein concentrations were adjusted to 6 mg/ml with the 2-D cell lysis buffer. Protein profiling was performed using two-dimensional difference gel electrophoresis (2-DIGE). Protein extracts from untreated and treated samples were labeled with different CyDye DIGE fluors (Cy5 untreated (red) and Cy3 treated samples (green). Pairs of untreated and treated samples were separated in the first-dimension strips pH 3 - 10 and then run on the 12% SDS gels. Gels were scanned on Typhoon TRIO (GE Healthcare) image scanner and analyzed with Image Quant software (GE Healthcare).

Protein spots were in-gel digested with trypsin, and samples were spotted onto the MALDI plate and analyzed by matrix-assisted laser desorption ionization-time-of-flight (MALDI/TOF) mass spectrometer (MS). GPS Explorer version 3.5 software was used to search the MASCOT database (Matrix science) to analyze MS and MS/MS spectra. The spots with significant statistical changes (p < 0.05) were determined by Student’s t-test. The top hit proteins were selected for identification of a spot. The mascot files and mass spec data had been uploaded into a public repository ProteomeXchange (ProteomeXchange Accession - PXD037194). In addition, the characteristics of identified proteins were performed with the Kyoto Encyclopedia of Genes and Genomes (KEGG) (https://www.kegg.jp/), The Arabidopsis Information Resource (TAIR) and UniProt (https://www.uniprot.org/).

### Measurement of ABA concentration

ABA concentration in Arabidopsis leaves was measured according to the method described by Kim et al. (Kim et al., 2009) with minor modifications. Briefly, about 150 treated seven day old Arabidopsis seedlings were ground into powder in liquid nitrogen and 1 ml of sterile water was added to the powdered plant material (200 mg). After overnight incubation at 4°C, the samples were centrifuged at 14,000 x g for 10 min. The supernatant was transferred to a new tube and lyophilized in a freeze-dryer. The dried material was dissolved in 60 μl of water. Five μl of the suspension was transferred to 95 μl of TBS buffer and subjected to ELISA bioassay (Phytodetek, Agdia, Inc.) according to the methods provided by the manufacturer.

### Statistical analysis

Results of the treatments for dose response assay were analyzed in R studio (version 3.4.1.) with the drc package. IC_50_ values for SPC and HERB were obtained using a four-parameter logistic function. Statistical analysis and graphs were generated with software GraphPad Prism 9.4.0

## Results

### The effects of spliceostatin C and herboxidiene on Arabidopsis seedlings

**Figure 2:**
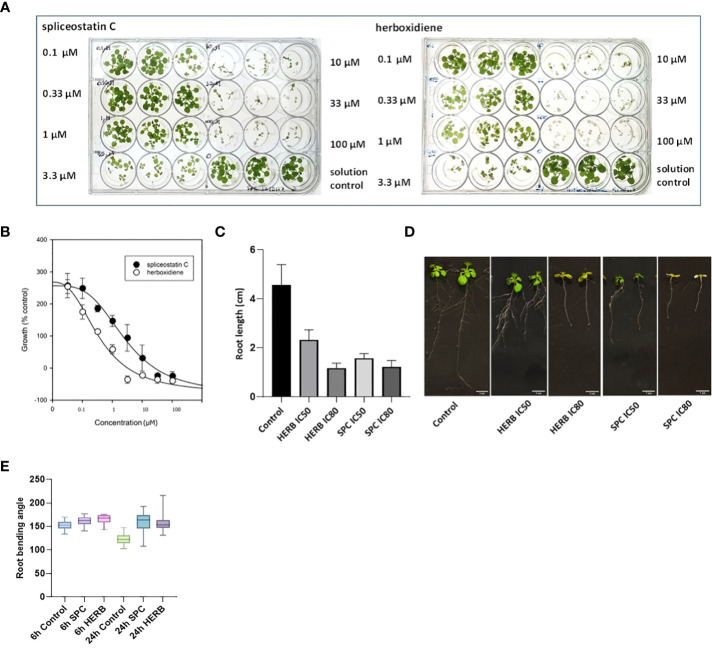
Dose-response of Arabidopsis seedlings treated with splicing inhibitors. Representative results are shown for spliceostatin C and herboxidiene seven days post-treatment **(A)**. Determination of IC_50_ and IC_80_ values. Data were collected 7 days after treatment of Arabidopsis seedlings. Data are shown as mean ± SD, (n=3) **(B)**. Growth inhibition of Arabidopsis seedlings by splicing inhibitors. Inhibition of root growth by SPC and HERB at concentrations of IC_50_ and IC_80_ **(C)**. Growth of Arabidopsis seedlings 7 days after treatment. Data are shown as mean ± SD, (n=20) **(D)**. Arabidopsis root gravitropism in response to the splicing inhibitors. See to SPC and HERB at the concentrations of IC_50_. Boxplot represents the distribution of root bending angles of curvature (n=20). Whiskers mark the minimum and maximum values **(E)**.

Spliceostatin C and herboxidiene, a structural analog of SPC (**Supplementary Figure 1**), strongly inhibited the growth of Arabidopsis seedlings. Leaves treated with concentrations of 10 μM SPC or 3.3 μM HERB exhibited extensive bleaching after exposure to the compounds (**Figure 2A**). The concentrations necessary to inhibit the leaf growth in 7-day-old seedlings by 50% (IC_50_) and 80% (IC_80_) were estimated at 2.2 µM and 15.2 µM for SPC and 0.37 µM and 1.72 µM for HERB, respectively (**Figure 2B**). Both inhibitors significantly reduced root growth and almost complete inhibited the lateral branching of roots (**Figures 2C, D**). Additionally, higher concentrations induced the development of reddish stems and leaves in some seedlings (**Supplementary Figure 3**). Moreover, the treated seedlings displayed a significant reduction in leaf size and stomata opening (**Supplementary Figures 4A–C**), as well as inhibition of gravitropism.

To further confirm the impact of SPC on the gravity-directed movement of root tips, seven-day-old Arabidopsis seedlings were exposed to 2.2 μM SPC and the plates were then turned 90 degrees. The root tips in control samples responded rapidly as compared to the treated samples (**Supplementary Figure 5**). The change in the angle of the root tips was observed 6 h after rotating the plates. Differences in tip angles between the control and the SPC treatment became greater after 24 h (**Figure 2E**). Most of SPC-treated seedlings had no significant adjustments in root tip curvature in response to changing the seedling orientation (**Supplementary Figure 5**; **Figure 2E**).

### Gene expression affected by spliceostatin C

**Figure 3:**
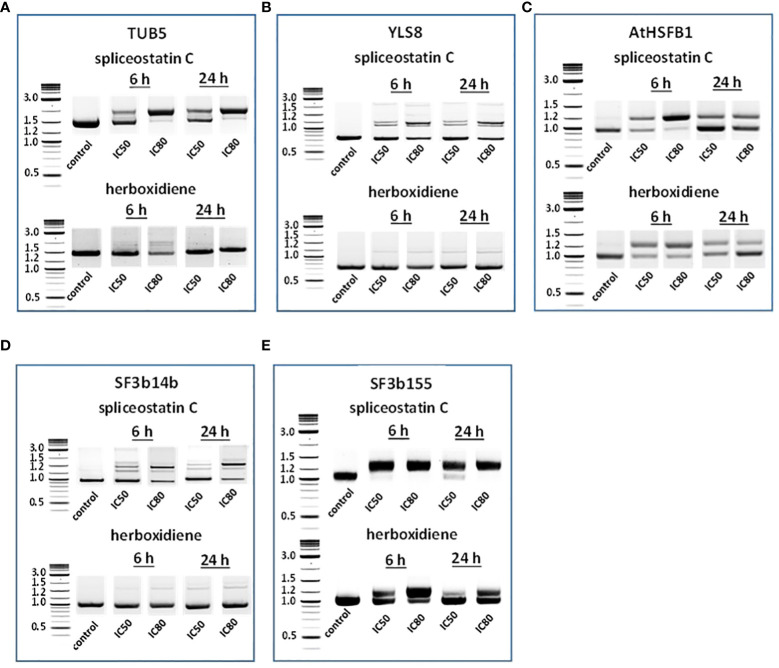
Detection of splicing activities by RT-sqPCR. The results demonstrate the maturation of precursor mRNA of selected genes affected by inhibitors leading to intron retention, alternative 5’ or 3’side splicing **(A–E)**. TUB5 **(A)**; YLS8 **(B)**; AtHSFB1 **(C)**; SF3b14b PHF5-like protein **(D)** and SF3b155 **(E)**.

**Figure 4:**
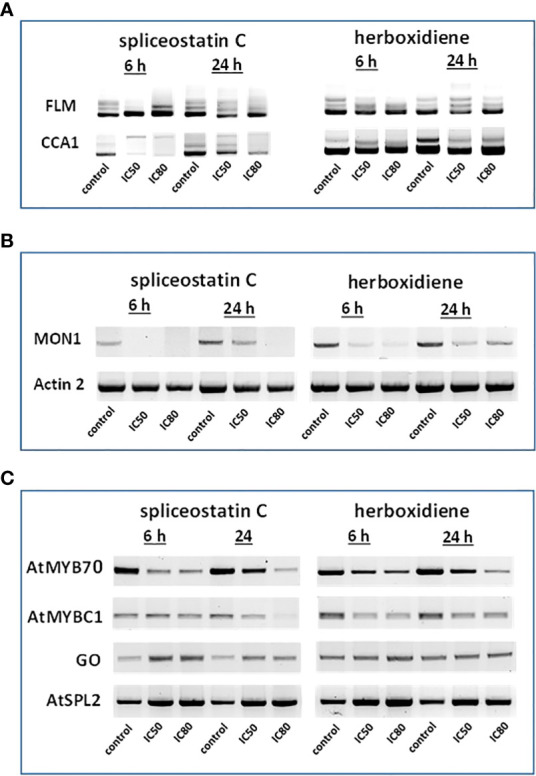
Effect of splicing inhibitors on the expression of selected genes. FLM and CCA1 **(A)**; MONENSIN SENSITIVITY1 (MON1) and actin 2 (ACT2) **(B)** and AtMYB70, AtMYBC1, GO (galactose oxidase) and AtSPL2 **(C)**.

To assess the impact of spliceostatin C on splicing, 19 genes were selected to monitor the length of their transcripts (**Supplementary Table 3**). These genes fall into four categories: 1) stably expressed genes that are used frequently as reference genes in the RT-qPCR method; 2) regulation factors of which the expression is regulated by AS which resulting in the appearance of multiple transcripts; 3) splicing factors; and 4) intronless genes. The effects of SPC and herboxidiene on these genes were investigated by RT-sqPCR (**Figure 3**) using the primers spanning intron. The strategy and examples of alternative splicing illustrated in (**Supplementary Figure 6**). As shown in **Figures 3A–E**, intron retention and AS occurred upon the exposure of the seedlings to the compounds at the concentrations of IC_50_ or IC_80_ for 6 and 24 h. Among these genes, seven transcripts underwent intron rearrangements such as intron retention and possible alternative 5’ or 3’ splicing site. They are SF3b14b PHF5-like protein, YLS8, SF3b155, TUB5, AtHSFB1, FLM and CCA1 (**Figures 3A–E**, **4A**). The expression of the genes in the ‘stable expressed’ group (**Supplementary Table 3**) was affected by both the splicing inhibitors in two ways: the decline in gene expression and/or the appearance of additional transcripts. The transcription of YLS8, TUB5 and AtHSFB1 resulted in at least one erroneous transcript (**Figures 3A–E**). To validate the RT-sqPCR results, the RT-sqPCR (**Supplementary Data Sheet 1**) products (un-spliced and correctly spliced) were sequenced to confirm the correct fragments observed on the agarose gels, examples shown in **Supplementary Figures 7–11**. Five bands in SPC-treated TUB5 samples were identified. The genomic sequence of TUB5 (1,983 bp) contains four introns, the longest fragment that retained all introns. Others retain one or two introns, 1892 bp and 1,781 bp, and the shortest transcript was a mature mRNA having 1,621 bp (**Figure 3A**). The transcription of YLS8 was also affected by SPC at the concentrations of IC_50_ and IC_80_. It caused the appearance of two additional transcripts, 1,167 and about 1,050 bp fragments, and the mature mRNA is 702 bp (**Figure 3B**). However, the splicing process of TUB5 and YLS8 was not as significantly affected by herboxidiene. In the case of AtHSFB1, which contains one intron only, an additional fragment by RT-sqPCR was a result of the retention of 192 bp intron (**Figure 3C**). At the concentration of IC_50_ and the 6 h time point, the treatments of SPC and HERB led to the accumulation of a comparable level of pre-mRNA, while at a higher concentration of SPC, i.e., IC_80_, the splicing process was almost completely inhibited (**Figure 3C**). SF3b14b and SF3b155 belong to splicing factors (**Supplementary Table 3**). In the case of SF3b14b, two additional fragments were observed when Arabidopsis seedlings were exposed to SPC, suggesting a result of intron retention (**Figure 3D**), while the splicing process was barely affected by herboxidiene. SF3b155 contains one intron only which is located at the 5’-UTR and its pre-mRNA extended to 4748 base pairs. To accommodate the PCR reactions, we designed primers that included the intron region and spanned a part of the coding sequence. The results showed that SPC almost completely inhibited the intron-splicing process at concentrations of IC_50_ and IC_80_ at both time points. In contrast, the intron-splicing was partially inhibited by herboxidiene under the same conditions (**Figure 3E**).

In *Arabidopsis thaliana*, there are two critical regulation factors, FLOWERING LOCUS M (FLM) and CIRCADIAN CLOCK ASSOCIATED 1 (CCA1). FLM is a critical flowering regulator and the CCA1 gene encodes an MYB-related transcription factor. The pre-mRNAs of FLM and CCA1 contain five and seven introns, respectively, which offered a wide range of possibilities for heterogeneity of transcripts (**Figure 4A**). Although the mature transcript, a 794 bp fragment without any intron, was the most abundant fragment in the control samples, five partially processed FLM were visible on agarose gels. As shown in **Figure 4A**, The exposure of Arabidopsis seedlings to SPC or herboxidiene for 6 h seems to promote the splicing process, i.e., less incompletely processed transcripts. Interestingly, this process was arrested when the exposure extended to 24 h. On the contrary, the expression of CCA1 was suppressed by SPC at an early time point, but not by HERB (**Figure 4A**).

The expression of MON1, which contains 13 introns, was suppressed by SPC and HERB at an early time point (**Figure 4B**). ACT2, the most frequently used as a reference gene for RT-qPCR in Arabidopsis (Czechowski et al., 2005), showed minimal but noticeable changes when the seedlings were exposed to the compounds in our experiment conditions (**Figure 4B**). In the intronless category (**Supplementary Table 3**), when treated with SPC or HERB, the expression of transcription factors AtMYB70 and AtMYBC1 decreased, while AtSPL2 and galactose oxidase slightly increased in response to the treatments (**Figure 4C**). Taken together, the RT-sqPCR results suggest that SPC and HERB distinct from one another although they are structurally similar.

Splicing inhibitors decreased the presence of a majority of other proteins featured in the **Supplementary Table 3**. except two that surprisingly had increased level of transcripts (GAPH from Stable expression and ATSPL Intronless groups, respectively). Only one gene ACT2 kept the same, stable level of expression (data not shown).

### Alterations of Arabidopsis proteome induced by spliceostatin C

**Figure 5:**
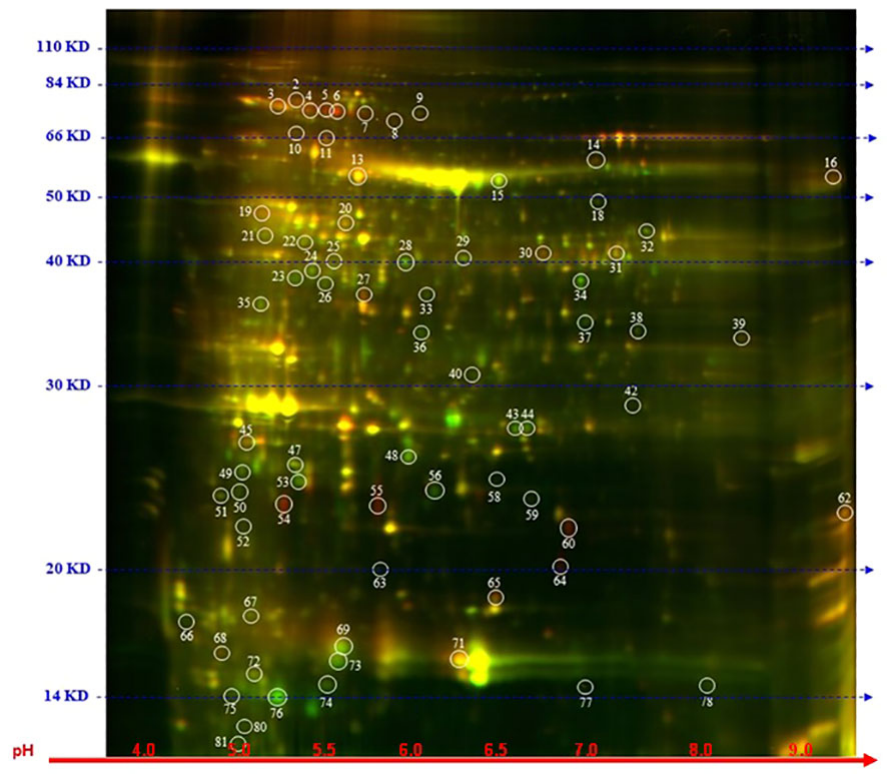
Distribution of differential proteins identified on 2D-DIGE gel. Proteins from untreated samples were labeled with Dy3 (green)and samples treated with SPC were labeled with Dy5 (red). Images were analyzed with Image Quant software to detect the differentially expressed protein spots, marked with circles (Student’s t-test, *p* ≤ 0.05). Numbers indicate spots that significantly differ at 48 h compared to the control.

To explore further the impact of the splicing inhibitors on protein translation, we performed proteomic profiling, which could help to understand the impact of SPC on the phenotype of treated seedlings. In this experiment, Arabidopsis seedlings were treated with SPC at the concentration of IC50 (2.2 μM) and incubated for 48 h in the growth chamber. Proteins were extracted and subjected to two-dimensional difference gel electrophoresis (2D-DIGE), the technique that would reveal any aberrant proteins generated during translation from abnormal mRNAs caused by spliceosome inhibition. Our results showed that upon SPC exposure the concentration of 66 proteins was significantly affected (Student’s t-test, p ≤ 0.05) with 38 of them decreased and 28 increased (**Figure 5**; **Table 1**). All sixty-six spots were further analyzed using MALDI/TOF spectrophotometry. The MS/MS ion search in the MASCOT database identified 40 unique proteins and 26 isoforms with the highest scores **Supplementary Table 4**).

**Table 1:** Proteins affected by IC_50_ SPC treatments.

Spot No.	Av. Ratio		p-value	Protein names	Localization	TAIR
64	11.36		0.0012	Extra-large guanine nucleotide-binding protein 2 (Extra-large GTP-binding protein 2) (Extra-large G-protein 2)	nucleus	AT4G34390
55	8.91		0.0018	Thylakoid lumenal protein TL20.3, chloroplastic	chloroplast	AT1G12250
54	5.18		0.002	17.4 kDa class I heat shock protein (17.4 kDa heat shock protein 1) (AtHsp17.4A)	cytoplasm	AT3G46230
60	5.04		0.0055	17.6 kDa class I heat shock protein 2 (17.6 kDa heat shock protein 2) (AtHsp17.6B)	cytoplasm	AT2G29500
6	5.01		0.0025	Heat shock 70 kDa protein 5 (Heat shock protein 70-5) (AtHsp70-5) (Heat shock protein 70b)	cytoplasm	AT1G16030
5	4.68		0.0092	Heat shock 70 kDa protein 5 (Heat shock protein 70-5) (AtHsp70-5) (Heat shock protein 70b)	cytoplasm	AT1G16030
4	2.72		0.016	Heat shock 70 kDa protein 5 (Heat shock protein 70-5) (AtHsp70-5) (Heat shock protein 70b)	cytoplasm	AT1G16030
8	2.47		0.042	Ribulose bisphosphate carboxylase large chain (RuBisCO large subunit) (EC 4.1.1.39)	chloroplast	ATCG00490
9	2.32		0.022	Ribulose bisphosphate carboxylase large chain (RuBisCO large subunit) (EC 4.1.1.39)	chloroplast	ATCG00490
7	2.24		0.00006	Ribulose bisphosphate carboxylase large chain (RuBisCO large subunit) (EC 4.1.1.39)	chloroplast	ATCG00490
27	2.15		0.0063	PYK10-binding protein 1 (Jacalin-related lectin 30) (Jasmonic acid-induced protein)	cytoplasm	AT3G16420
65	2.02		0.0012	Ribulose bisphosphate carboxylase small chain 2B, chloroplastic OS=Arabidopsis thaliana OX=3702 GN=	chloroplast	AT5G38420
2	1.96		0.016	Heat shock 70 kDa protein BIP1 (Heat shock 70 kDa protein 11) (Heat shock protein 70-11) (AtHsp70-11) (Luminal-binding protein 1) (AtBP1) (BiP1)	endoplasmic reticulum lumen/nucleus	AT5G28540
68	1.94		0.00063	Ribulose bisphosphate carboxylase small chain 2B, chloroplastic (RuBisCO small subunit 2B) (EC 4.1.1.39)	chloroplast	AT5G38420
10	1.86		0.0034	Chaperonin 60 subunit alpha 1, chloroplastic (CPN-60 alpha 1) (Protein SCHLEPPERLESS) (RuBisCO large subunit-binding protein subunit alpha 1)	chloroplast	AT2G28000
11	1.83		0.0037	ATP synthase subunit alpha, chloroplastic (EC 7.1.2.2) (ATP synthase F1 sector subunit alpha) (F-ATPase subunit alpha)	chloroplast	ATCG00120
45	1.83		0.046	2-Cys peroxiredoxin BAS1, chloroplastic (2-Cys Prx A) (2-Cys peroxiredoxin A) (EC 1.11.1.24) (Thiol-specific antioxidant protein A) (Thioredoxin-dependent peroxiredoxin BAS1)	chloroplast	AT3G11630
3	1.79		0.019	Heat shock 70 kDa protein 1 (Heat shock cognate 70 kDa protein 1) (Heat shock cognate protein 70-1) (AtHsc70-1) (Heat shock protein 70-1) (AtHsp70-1) (Protein EARLY-RESPONSIVE TO DEHYDRATION 2)	cytosol/nucleus	AT5G02500
71	1.75		0.0043	Ribulose bisphosphate carboxylase small chain 2B, chloroplastic (RuBisCO small subunit 2B) (EC 4.1.1.39)	chloroplast	AT5G38420
39	1.71		0.0087	Lectin-like protein LEC (AtLEC) (Ath.lec2)	apoplast	AT3G15356
62	1.68		0.005	Photosystem I reaction center subunit II-2, chloroplastic (Photosystem I 20 kDa subunit 2) (PSI-D2)	chloroplast	AT1G03130
20	1.64		0.0024	Actin-8	cytoskeleton	AT1G49240
30	1.64		0.00016	Glyceraldehyde-3-phosphate dehydrogenase GAPC1, cytosolic (EC 1.2.1.12) (NAD-dependent glyceraldehydephosphate dehydrogenase C subunit 1)	nucleus	AT3G04120
13	1.57		0.048	ATP synthase subunit beta, chloroplastic (EC 7.1.2.2) (ATP synthase F1 sector subunit beta) (F-ATPase subunit beta)	chloroplast	ATCG00480
14	1.57		0.0016	Serine hydroxymethyltransferase 1, mitochondrial (AtSHMT1) (EC 2.1.2.1) (Glycine hydroxymethyltransferase 1) (Serine Transhydroxymethyltransferase) (STM) (Serine methylase 1)	mitochondrion	AT4G37930
16	1.55		0.0072	Elongation factor 1-alpha 2 (EF-1-alpha 2) (eEF-1A2)	cytoplasm	AT1G07930
19	1.53		0.0076	Glutamine synthetase, chloroplastic/mitochondrial (EC 6.3.1.2) (GS2) (Glutamate–ammonia ligase)	chloroplast, mitochondrion	AT5G35630
31	1.52		0.0062	Glyceraldehyde-3-phosphate dehydrogenase GAPC1, cytosolic (EC 1.2.1.12) (NAD-dependent glyceraldehydephosphate dehydrogenase C subunit 1)	nucleus	AT3G04120
21	-1.44		0.023	Phosphoglycerate kinase 2, chloroplastic (EC 2.7.2.3)	chloroplast	AT1G56190
32	-1.53		0.028	Beta-glucosidase 23 (AtBGLU23) (EC 3.2.1.21) (Protein PHOSPHATE STARVATION-RESPONSE 3.1)	Endoplasmic reticulum lumen	AT3G09260
72	-1.53		0.034	Thioredoxin M2, chloroplastic (AtTrxm2)	chloroplast	AT4G03520
67	-1.55		0.012	Ribulose bisphosphate carboxylase small chain 1A, chloroplastic (RuBisCO small subunit 1A) (EC 4.1.1.39)	chloroplast	AT1G67090
47	-1.57		0.011	2-Cys peroxiredoxin BAS1, chloroplastic (2-Cys Prx A) (2-Cys peroxiredoxin A) (EC 1.11.1.24) (Thiol-specific antioxidant protein A) (Thioredoxin-dependent peroxiredoxin BAS1)	chloroplast	AT3G11630
69	-1.59		0.027	Ribulose bisphosphate carboxylase small chain 2B, chloroplastic (RuBisCO small subunit 2B) (EC 4.1.1.39)	chloroplast	AT5G38420
15	-1.6		0.036	Ribulose bisphosphate carboxylase large chain (RuBisCO large subunit) (EC 4.1.1.39)	chloroplast	ATCG00490
22	-1.62		0.016	Ribulose bisphosphate carboxylase/oxygenase activase, chloroplastic (RA) (RuBisCO activase)	chloroplast	AT2G39730
58	-1.63		0.041	Ribulose bisphosphate carboxylase large chain (RuBisCO large subunit) (EC 4.1.1.39)	chloroplast	ATCG00490
33	-1.64		0.0034	Putative WEB family protein At1g65010, chloroplastic	chloroplast	AT1G65010
76	-1.64		0.0045	Ribulose bisphosphate carboxylase small chain 2B, chloroplastic (RuBisCO small subunit 2B) (EC 4.1.1.39)	chloroplast	AT5G38420
24	-1.65		0.018	Fructose-bisphosphate aldolase 2, chloroplastic (AtFBA2) (EC 4.1.2.13)	chloroplast	AT4G38970
38	-1.67		0.0056	Probable xyloglucan endotransglucosylase/hydrolase protein 7 (At-XTH7) (XTH-7) (EC 2.4.1.207)	cell wall, apoplast	AT4G37800
18	-1.7		0.025	Catalase-2 (EC 1.11.1.6)	peroxisome, glyoxysome, cytoplasm	AT4G35090
78	-1.7		0.0078	Ribulose bisphosphate carboxylase small chain 1A, chloroplastic (RuBisCO small subunit 1A) (EC 4.1.1.39)	chloroplast	AT1G67090
73	-1.78		0.0035	Ribulose bisphosphate carboxylase small chain 1A, chloroplastic (RuBisCO small subunit 1A) (EC 4.1.1.39)	chloroplast	AT1G67090
74	-1.78		0.0068	Ribulose bisphosphate carboxylase small chain 1A, chloroplastic (RuBisCO small subunit 1A) (EC 4.1.1.39)	chloroplast	AT1G67090
51	-1.83		0.0027	2-Cys peroxiredoxin BAS1, chloroplastic (2-Cys Prx A) (2-Cys peroxiredoxin A) (EC 1.11.1.24) (Thiol-specific antioxidant protein A) (Thioredoxin-dependent peroxiredoxin BAS1)	chloroplast	AT3G11630
63	-1.86		0.047	Protein RALF-like 32	apoplast	AT4G14010
37	-1.97		0.01	Ribulose bisphosphate carboxylase large chain (RuBisCO large subunit) (EC 4.1.1.39)	chloroplast	ATCG00490
35	-1.98		0.017	ATP synthase subunit alpha, chloroplastic (EC 7.1.2.2) (ATP synthase F1 sector subunit alpha) (F-ATPase subunit alpha)	chloroplast	ATCG00120
44	-1.98		0.015	Beta carbonic anhydrase 1, chloroplastic (AtbCA1) (AtbetaCA1) (EC 4.2.1.1) (Beta carbonate dehydratase 1) (Protein SALICYLIC ACID-BINDING PROTEIN 3) (AtSABP3)	cell membrane, chloroplast stroma	AT3G01500
75	-1.98		0.0012	Profilin-2 (AtPROF2) (AthPRF2)	cytoskeleton, reticulum, nucleus, cytoplasm	AT4G29350
77	-1.98		0.015	Chlorophyll a-b binding protein 3, chloroplastic (Chlorophyll a-b protein 180) (CAB-180) (LHCII type I CAB-3)	chloroplast	AT1G29910
80	-1.98		0.0064	Photosystem II reaction center protein H (PSII-H) (Photosystem II 10 kDa phosphoprotein)	chloroplast	ATCG00710
25	-2.02		0.031	Ribulose bisphosphate carboxylase/oxygenase activase, chloroplastic (RA) (RuBisCO activase)	chloroplast	AT2G39730
36	-2.06		0.026	Ribulose bisphosphate carboxylase large chain (RuBisCO large subunit) (EC 4.1.1.39)	chloroplast	ATCG00490
81	-2.06		0.036	Cytochrome b559 subunit alpha (PSII reaction center subunit V)	chloroplast	ATCG00580
52	-2.1		0.0068	2-Cys peroxiredoxin BAS1, chloroplastic (2-Cys Prx A) (2-Cys peroxiredoxin A) (EC 1.11.1.24) (Thiol-specific antioxidant protein A) (Thioredoxin-dependent peroxiredoxin BAS1)	chloroplast	AT3G11630
23	-2.17		0.031	Fructose-bisphosphate aldolase 1, chloroplastic (AtFBA1) (EC 4.1.2.13)	chloroplast	AT2G21330
42	-2.21		0.022	Beta carbonic anhydrase 1, chloroplastic (AtbCA1) (AtbetaCA1) (EC 4.2.1.1) (Beta carbonate dehydratase 1) (Protein SALICYLIC ACID-BINDING PROTEIN 3) (AtSABP3)	cell membrane, chloroplast stroma	AT3G01500
40	-2.37		0.012	ATP synthase subunit beta, chloroplastic (EC 7.1.2.2) (ATP synthase F1 sector subunit beta) (F-ATPase subunit beta)	chloroplast	ATCG00480
56	-2.38		0.021	Thylakoid lumenal 19 kDa protein, chloroplastic (P19)	chloroplast	AT3G63540
66	-2.46		0.011	PLAT domain-containing protein 2 (AtPLAT2) (PLAT domain protein 2)		AT2G22170
43	-2.47		0.053	Beta carbonic anhydrase 1, chloroplastic (AtbCA1) (AtbetaCA1) (EC 4.2.1.1) (Beta carbonate dehydratase 1) (Protein SALICYLIC ACID-BINDING PROTEIN 3) (AtSABP3)	cell membrane, chloroplast stroma	AT3G01500
34	-2.48		0.019	Beta-glucosidase 23 (AtBGLU23) (EC 3.2.1.21) (Protein PHOSPHATE STARVATION-RESPONSE 3.1)	Endoplasmic reticulum lumen	AT3G09260
53	-2.64		0.034	Ribulose bisphosphate carboxylase large chain (RuBisCO large subunit) (EC 4.1.1.39)	chloroplast	ATCG00490
59	-3.48		0.032	12S seed storage protein CRB (Cruciferin 2) (AtCRU2) (Cruciferin B) (Legumin-type globulin storage protein CRU2)	vacuole	AT1G03880

The most upregulated proteins and their isoforms preserved their original molecular weight with some shifts due to posttranslational modifications and/or imperfection of the 2D gel technique. The molecular weight of five proteins observed on the 2D-gels was increased by at least 10 kDa (**Figure 5**, spots # 7, 8, 9,11 and 16) and two of them migrated faster (**Figure 5**, spots # 55 and 64). The concentration of these two proteins (spots) increased the most among all proteins. The average ratio (treated versus control group) of extra-large guanine nucleotide-binding protein (XLG2, AT4G34390) and thylakoid lumen protein (TL20.3, AT1G12250) reached 11.36 and 8.91, respectively. Among 38 downregulated isoforms (spots), at least 15 of them (spots # 25, 32, 33, 34, 35, 36, 37, 40, 43, 44, 53, 58, 59 and 77) were observed with gel mobility shifted, suggesting their lower MW than originally calculated. These results indicate that the concentration of these identified degradation products increased after SPC treatment due to a possible malfunction of proteolytic processes.

### Impact of splicing inhibitors on hormone management

**Figure 6:**
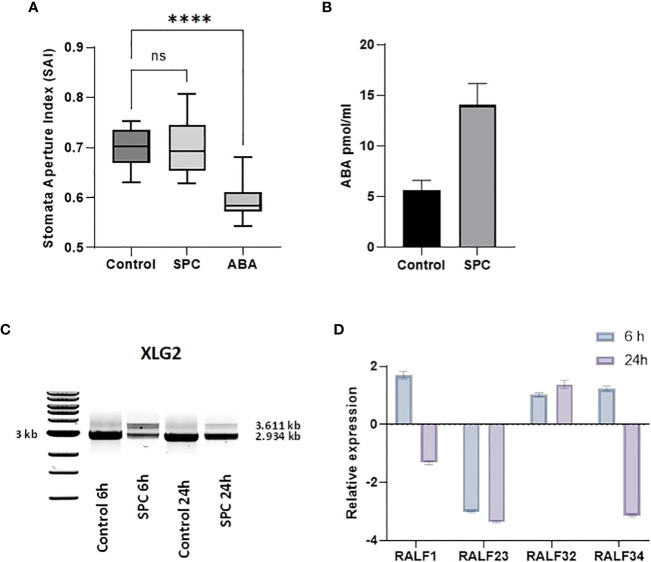
Impact of SPC on stomata aperture and the involvement of ABA signaling. Stomata aperture of Arabidopsis leaves (n=20) was measured in various conditions (see Materials and Methods). Data were analyzed with one-way ANOVA multiple comparisons to confirm the significant difference between control and SPC-treated samples versus ABA-treated samples; ns, no significance; symbol **** means statistically significant difference between treatments (p < 0.0001) **(A)**. ABA concentrations were measured 6 h post treatment of 7 day old Arabidopsis seedlings SPC-treated and control tissues. Data are shown as mean ± SD, (n=3) **(B)**. Effect of spliceostatin C on the expression of XLG2. Samples were collected at two-time points (6 and 24 h) after exposure to 2.2 µM of SPC. RNA was extracted and used for RT-sqPCR analysis **(C)**. Expression of RALF genes in response to SPC. Four RALF genes (RALF1, RALF23, RALF 32 and RALF34) were examined by quantitative reverse transcription-polymerase chain reaction (RT-qPCR) for responsiveness to SPC. Assays were performed in triplicates. Data are shown as mean ± S.D **(D)**.

Stomatal aperture is regulated by various exogenous stimuli. These stimuli are sensed and signaled to the guard cells *via* signaling molecules. Abscisic acid (ABA) is among the major players (Munemasa et al., 2015) and the main hormone responsible for closing stomata at night as well as responding to environmental conditions. It has been reported that splicing inhibitors prevent the closing of stomata (Alshareef et al., 2017; Ling et al., 2017). To determine whether SPC also stimulates similar reactions, we measured the stomatal apertures by direct microscopic observation (see Materials and Methods). The stomatal apertures from leaf tissues treated with SPC at a concentration of IC_80_ did not show a difference from those of control; however, it responded to ABA, which was consistent with the previous report (Acharya and Assmann, 2009) (**Figure 6A**; **Supplementary Figure 4**).

To explore further whether SPC is involved in the regulation of ABA, we performed an ELISA bioassay to determine the concentration of the hormone in SPC-treated samples. As shown in **Figure 6B**, the concentration of ABA in the SPC-treated tissues increased 2.4 times compared to the control (6 h after exposure), suggesting that the splicing inhibitor SPC may have impacted ABA-related response by interfering with the ABA biosynthetic pathway or impairing ABA transport to guard cells. A Survey of our proteomic data revealed that five proteins were associated with ABA-dependent signaling or guard cell movement (**Table 1**). They are 12S seed storage protein CRB (AtCRU2) (Lawrence et al., 2020), beta carbonic anhydrase 1 (AtbCA1) (Clement et al., 2011), heat shock 70 kDa protein 1 (AtHsc70-1) (Clement et al., 2011), rapid alkalinization factor (RALF)-like 32, and extra-large guanine GTP-binding protein 2 (XLG2). Among these proteins, RALF-like 32 and XLG2 are putative components in signal transduction systems involved in stomatal aperture control (Yu et al., 2018).

The level of XLG2 truncated protein was significantly elevated in SPC-treated samples as shown in the proteomics data (**Table 1**). To look into the details of the gene expression and the corresponding protein content, we carried out RT-sqPCR analyses. The gene coding for XLG2 contains seven small introns between 92 and 113 bps. Primers were designed to include the 5’and 3’ UTRs. The full-length PCR product (mature mRNA) was predicted 2,934 bp in length. The amplicon length in treated samples is similar to those in control samples, but their intensity differs significantly (**Figure 6C**). Two additional fragments were observed that may represent the transcripts that were not fully spliced, and their intensity also varies significantly, indicating that SPC may affect the splicing process.

Rapid alkalinization factors (RALF) are cysteine-rich small proteins typically having 80–120 amino acids (Campbell and Turner, 2017). As mentioned above, our proteomics data showed that the level of RALF-like 32, a protein associated with ABA-dependent signaling and calcium-mediated signaling, decreased after SPC treatment. RALF-like 32 was placed in clade III, one of the four major clades (Campbell and Turner, 2017). To assess the gene expression that may be potentially affected by the SPC, the RT-qPCR technique was used in this study. Since the coding sequence does not contain intron so the expression level would not be directly affected by the splicing inhibitor. To assess other members of the RALF protein family in response to SPC in Arabidopsis, we included three additional RALFs: RALF1 (AT1G02900), RALF23 (AT3G16570), and RALF34 (AT5G67070) in the experiments. Our results showed that all these three transcripts were down-regulated at 24-hour timepoint except RALF-like 32 (**Figure 6D**) that was down-regulated in the proteomics data (**Table 1**). The discrepancy between proteomics data and results of the RT-qPCR remains unknown.

### Molecular modeling suggests the possible binding site of spliceostatin C in spliceosome

**Figure 7:**
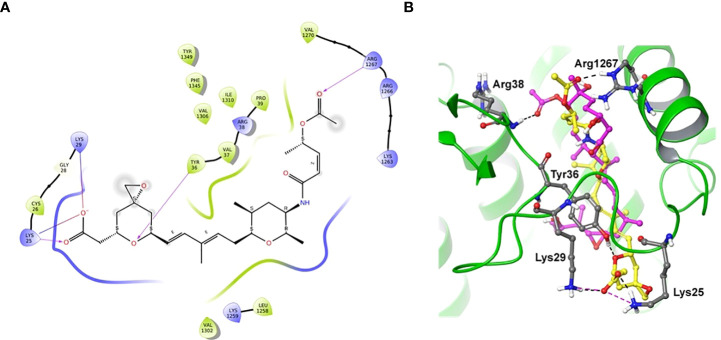
Two-dimensional interaction diagram and overlaid representation of spliceostatin C and pladienolide B with a homology model of SF3B1-PHF5A. Overlay of SPC (carbon in yellow) and pladienolide B (magenta) with homology model of SF3B1-PHF5A subunits of *A*. *thaliana* **(A)**; The docking results showed that spliceostatin C formed H-bonding with Arg1267 and Tyr36, which are critical residues for herbicidal activity. In addition, it forms salt-bridges with Lys 25 and 29. Overall, the spliceostatin C occupied the same site where pladienolide B binds in SF3B1-PHF5A subunits **(B)**.

Docking of spliceostatin C into the homology model of SF3B1-PHF5A subunits of Arabidopsis thaliana was conducted to understand the putative binding site and its interactions profile. Since an X-ray crystal structure is unavailable for SF3B1-PHF5A subunits of *Arabidopsis thaliana*, a homology model was constructed using the X-ray crystal structure of a human SF3B core complexed with pladienolide B as a template (PDB Code: 6EN4) (Cretu et al., 2018). The docking results showed that SPC formed H-bonding with Tyr36 and Arg1267 (**Figure 7**), which are the critical residues for herbicidal activity. In addition, it formed salt-bridge interactions with Lys25 and Lys 29 (**Figure 7A**). Spliceostatin C occupied the same binding site as Pladienolide B in SF3B1-PHF5A subunits, yet, while the L-shaped distal half (tetrahydropyran group to acetylated ester segment) follows a common binding path in both ligands, the proximal half of SPC (epoxy-tetrahydropyran to a carboxyl group) is oriented in the opposite direction.

To investigate and understand the importance of the aromatic residue Y36 for Spliceostatin C binding, computational mutations with various amino acid residues (Y36L, Y36W, Y36A, Y36C, Y36R, and Y36E) were performed using the homology model of SF3B1-PHF5A subunits (**Supplementary Table 5**). Results indicate that the introduction of a charged amino acid at this position correlates to poorer binding free energy, which indicates reduced binding affinity of SPC with the mutated model. Specifically, when Y36 was mutated to glutamic acid (E), a bulky residue with a negative charge, the poorest binding free energy was observed, whereas, when the residue was mutated to a positively charged arginine (R), the decrease in binding free energy lessened (**Supplementary Table 5**). Additionally, mutation of Y36 to smaller residues, alanine (A) and cysteine (C), exhibited poorer binding free-energy compared to wild-type, suggesting the importance of an aromatic side chain at this position for spliceostatin C binding. Further, tryptophan (W), a larger residue, was also substituted for Y36 to validate the abovementioned finding. This substitution did not change the binding free energy of spliceostatin C with the corresponding mutated homology model of SF3B1-PHF5A compared to the wild-type, indicating the requirement for a bulky residue. The computationally predicted mutational results are further agreement with Teng et al. (Teng et al., 2017) Western blotting data. These findings suggest that the aromatic group is required for strong binding affinity while smaller residues or charged residues may negatively impact SPC binding with SF3B1-PHF5A subunits.

## Discussion

In the past, the role of introns and the process of their removal from RNA by spliceosomal catalysis had been greatly underestimated. Most eukaryotic genes contain at least one intron. Many researchers wondered why eukaryotic organisms use so much energy to tediously process RNA, and to prepare it for protein translation. Indeed, the entire mechanism of the intron excision, almost byzantine, involves hundreds of proteins. Such a complicated process should have a strong evolutionary purpose. Recently, alternative splicing of pre-mRNA to mature RNA was found to be a critical player in the control of gene expression. *In planta*, such post-transcriptional modifications are a crucial part of their very prompt and efficient adjustment to environmental changes like temperature and water availability. Thus, the plant cell tailors pre-mRNA to meet its specific needs. Clearly, AS-related regulation of gene expression plays a vital role in plant growth, development, and response to environmental factors.

Spliceostatins are well-known splicing inhibitors, and their inhibitory activity has been tested and studied on numerous mammalian models throughout the years (Corrionero et al., 2011; Teng et al., 2017). Nonetheless, their exact mode of action *in planta* and their phytotoxic effects are still relatively understudied. In this study, we examined the effect of SPC on selected transcripts and the entire proteome of Arabidopsis seedlings. To our knowledge, this is the first report that covers the impact of splicing inhibition on the entire plant proteome. We decided to use herboxidiene as a positive control since it is a splicing inhibitor with confirmed herbicidal activity (Miller-Wideman et al., 1992). The post-emergence test against Arabidopsis seedlings growing on phytagel demonstrated a strong phytotoxic activity of spliceostatin C, although the IC_50_ value was six times greater than that of herboxidiene.

### SPC impacts mRNA processing

Even though our representative sample of nineteen genes selected for RT-sqPCR was relatively small compared to recent RNA seq experiments testing splicing inhibitory properties of pladienolide B and herboxidiene (Alshareef et al., 2017; Ling et al., 2017), our results were similar. The highest number and concentration of aberrant transcripts were found at the early time point (6 h), while the original transcript pattern seems to slowly recover after 24 h. The comparison of this set of transcripts to other ones displaying the original transcript size and a strong up or down-regulation throughout all time points would suggest a weakening of the inhibitor effect directly on the spliceosome. This phenomenon could be due to the loss of the inhibitor *in vivo* by its degradation. An interesting example in this RT-sqPCR experiment is FLOWERING LOCUS M (FLM) gene. The FLM gene is part of the system controlling flowering in a temperature-dependent manner. The foundation of regulating its expression is AS that simultaneously produces several transcripts. As shown in **Figure 4**, SPC reduced the number of AS events in line with previously mentioned reports showing reduced AS processing across the entire transcriptome. Our results clearly demonstrated that SPC influenced the processing of pre-mRNA by alternative side splicing (**Figures 3**, **4**). Resent research indicates that spliceostatin A misleads the branch site (BS) recognition system which scans the intron for the next, most BS-resembling sequence (Cretu et al., 2021). In this study, the splicing of several but not all genes were affected by SPC and HERB. According to recent studies, the mechanism underlying such selectivity is extremely complex and largely depends on intron length and sequences of intron element BS and polypyrimidine tract (PPT) (Vigevani et al., 2017).

### Proteomics data revealed the involvement of signaling pathway in response to SPC

SPC treatment induced severe and diverse symptoms – starting from root growth inhibition (including lateral roots), leaf chlorosis and their development arrest, as well as root gravitropism and stomata impairment. These phenotypic effects are reflected in our proteome study. A large fraction of affected proteins were localized in the chloroplast, which is a signature indicator of plant stress that usually is induced by sensing changing environmental conditions (Zhu, 2016). Based on the pool of affected proteins, it can be assumed that SPC treatment induced a response that resembles the response to abiotic stresses such as ABA-dependent, heat and drought stresses. Our proteome results are consistent with two other reports on the effect of splicing inhibitors on Arabidopsis seedlings (Alshareef et al., 2017; Ling et al., 2017), even though their data were obtained from RNA-seq experiments. During stress, plants activate chloroplast proteolytic machinery to eliminate damaged chloroplast elements (Yang et al., 2019). A group of 15 proteins displayed lower molecular weights than expected might be due to such proteolysis triggered by SPC.

The presumption is that SPC and HERB have only one molecular target as phytotoxins. Thus, all that occurs after this primary effect are secondary effects of splicing inhibition. Some of these effects are due to the activation of defenses to a phytotoxic chemical. Many sensing mechanisms responsible for plant acclimation are based on AS since such responses need to occur fast to save the plant from damage. Alteration of these responses will exacerbate the harmful effects of the splicing inhibitors. Abscisic acid, a key hormone in abiotic responses elevated concentration almost 2.5 times in the presence of SPC.

Interestingly, despite the fact that ABA controls stomata aperture (Daszkowska-Golec and Szarejko, 2013), stomata in our experiment remained open (**Figure 6A**). These results are in contrast with the previously presented data on splicing inhibitors where pladienolide B and HERB caused the closure of stomata. In this context, we decided to conduct a more detailed evaluation of stomata-related proteins affected by SPC. In our proteomic experiment, a spot with the highest ratio contained peptides belonging to the extra-large guanine GTP-binding protein 2 (XLG2; spot # 64, **Figure 5**; **Table 1**). The spot migrated in the 2 D gel to the place of a 20 kDa protein, about 70 kDa less than a mature protein. It is difficult to determine, based on the dispersion of peptides from trypsin digestion, along with amino acid sequences whether the spot is a product of erroneous splicing or proteolysis. Considering these results, the types of XLG2 transcripts occurring in control and treated samples were assessed. Control samples contained two additional transcripts besides the main one with a correct length (not reported before). In 6 h of treatment, SPC elevated the amount of these two additional and reduced the correct transcripts. As with other examples in this experiment, we observed that transcripts are returning to their normal state at the 24 h time point. Again, this may be due to *in vivo* degradation of the splicing inhibitors.

Due to the positioning of multiple Leucine-Rich Repeats Receptor-Like Kinases (LRR-RKs) and Leucine-Rich Repeats Receptor-Like Proteins (LRP-RPs) along the plasma membrane, the plant is able to sense the environment and maintain communication between cells. This capability depends on the presence of various protein-protein interaction domains creating complex signal transduction multilevel modules (Dufayard et al., 2017). XLG2 is one of many proteins involved in such signal transduction process. According to multiple reports, XLG2 is an irreplaceable and multitasking regulatory protein in response to stress (e.g., XLG2 mutants are more sensitive to *Pseudomonas syringae* infection), stomata aperture, root morphology, etc. (Zhu et al., 2009; Maruta et al., 2015). Since it functions as a molecular switch, its expression and thereby presence must be very carefully managed. Zhu and coauthors proved that XLG2 is a target of extensive proteasome-mediated protein degradation, and detection of its functional form is extremely difficult (Zhu et al., 2009). Perhaps we observed results of such effective proteolysis in our study. As an element of signal transduction, XLG2 possibly also regulates AS of genes encoding stress-related proteins which may explain its localization in both the nucleus and plasma membrane (Maruta et al., 2015). Another part of the signaling system related to our results from this study is a downregulated peptide RALF32. The Arabidopsis RALF family consists of about 39 members that play multiple roles in plant development and stress responses (Abarca et al., 2021). RALF1 is one of the most well-researched polypeptides of its family (e.g., it promotes stomatal closure and inhibits opening stomata), and most RALFs inhibit plant growth and even impact protein synthesis by phosphorylation of elF4E1, eukaryotic translation initiation factor (Yu et al., 2018; Zhu et al., 2020). In our study, at the 24-h time point, peptides RALF1, RALF23 and RALF 34 declined in expression, while RALF32 was slightly elevated (**Figure 6D**). The decreased expression of RALF1 might be one of the factors associated with the loss of control of stomata aperture observed in this experiment.

### Molecular docking predicts the potential targets of SF3B1-PHF5A subunits

To understand the specifics of the putative binding site and its interactions profile, we performed molecular docking of SPC into the homology model of SF3B1-PHF5A subunits of *A. thaliana*. The docking results showed that spliceostatin C formed H-bonding with Arg1267 and Tyr36, which are critical residues for herbicidal activity. In addition, it forms salt-bridges with Lys 25 and 29. Interestingly, during the preparation of this manuscript, the cryo-electron microscopy structure of a cross-exon prespliceosome-like complex arrested with spliceostatin A was released (Cretu et al., 2021). The comparisons of the docked pose of SPC and spliceostatin A in SF3B1-PHF5A subunits revealed that our binding pose (L-shaped) is very similar, including the proximal half of spliceostatin C with spliceostatin A. Overall, spliceostatin C occupied the same site where pladienolide B binds in SF3B1-PHF5A subunits.

The yield of crops depends mainly on factors such as climate conditions and pest species. Weeds are a major class of crop pests, which can drastically reduce or eliminate crop production if left uncontrolled. Thus, by volume, farmers use more herbicides than any other pesticide class. This has caused massive selection for the evolution of weeds resistant to almost every commercial herbicide class. As a result, there is a large and growing need for herbicides with new modes of action that are effective against current herbicide-resistant weeds (Gaines et al., 2020; Duke and Dayan, 2021). As mentioned in the introduction, SPC, along with romidepsin, is a component of a bioherbicide that is under development (Martin et al., 2021). A bioherbicide with two active ingredients, each with a novel mode of action (inhibition histone deacetylase (Owens et al., 2020) and spliceosome function) would make it very difficult for target weeds to evolve either target site or non-target site resistance (Gaines et al., 2020; Gressel, 2020). There are no weed management products on the market with such an attribute.

## Data availability statement

The proteomic data presented in the study are deposited in the ProteomeXchange repository, accession number PXD037194.

## Author contributions

JB-H, LB, RA and SD conceptualized the research. JB-H and ZP designed the research. JB-H, PP, ZP and AC conducted the experiments and analyzed the data. JB-H, MM and ZP wrote the manuscript. All authors contributed to the article and approved the submitted version.
