# Supplementary figures and images for: Spliceostatin C, a component of a microbial bioherbicide, is a potent phytotoxin that inhibits the spliceosome
Source: Front Plant Sci. 2023 Jan 12;13:1019938. doi: 10.3389/fpls.2022.1019938 (PMC9878571; doi:10.3389/fpls.2022.1019938)

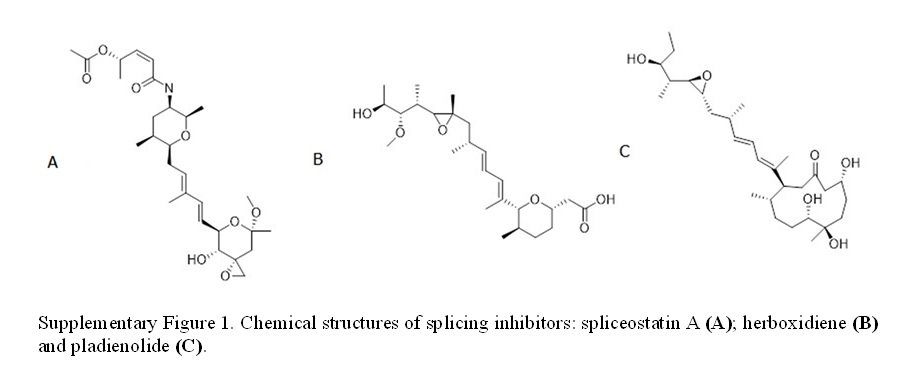

Supplement: Supplementary file 1 [file Image_1.jpg]

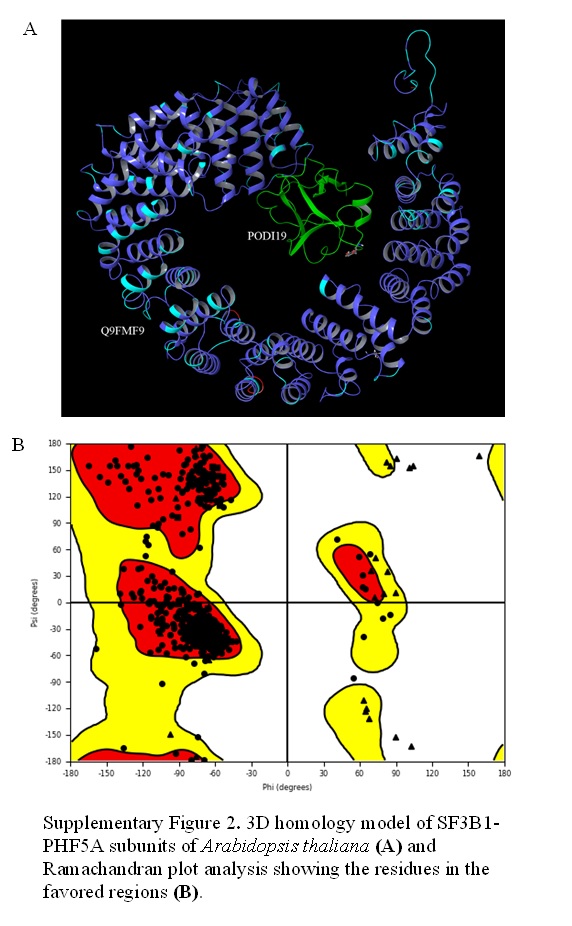

Supplement: Supplementary file 2 [file Image_2.jpg]

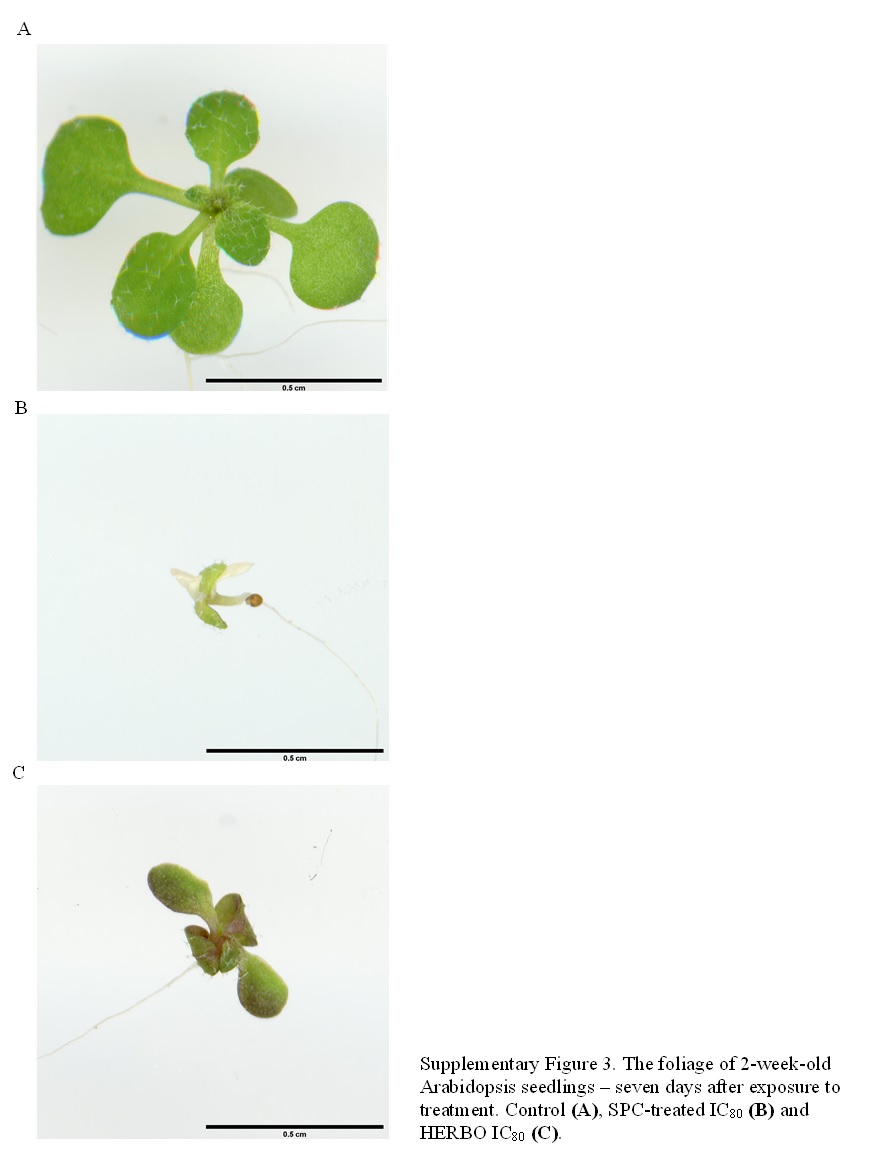

Supplement: Supplementary file 3 [file Image_3.jpg]

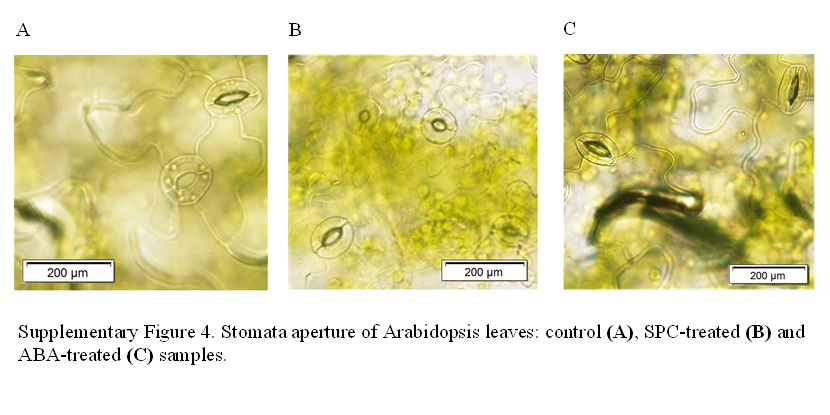

Supplement: Supplementary file 4 [file Image_4.jpg]

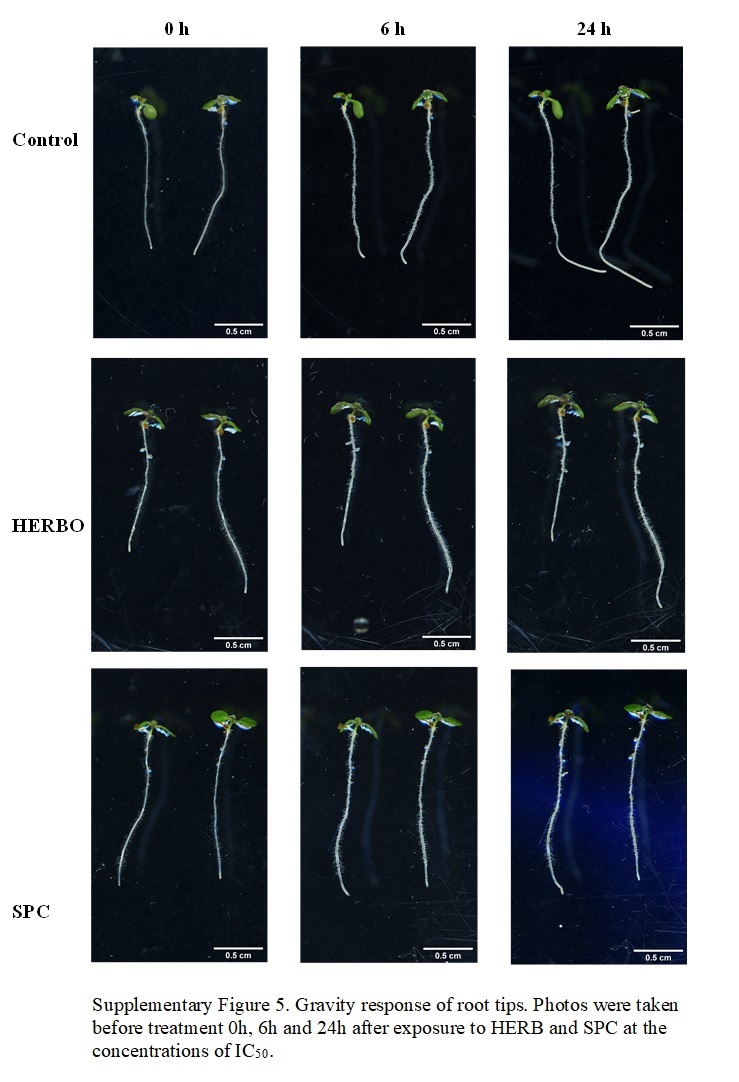

Supplement: Supplementary file 5 [file Image_5.jpg]

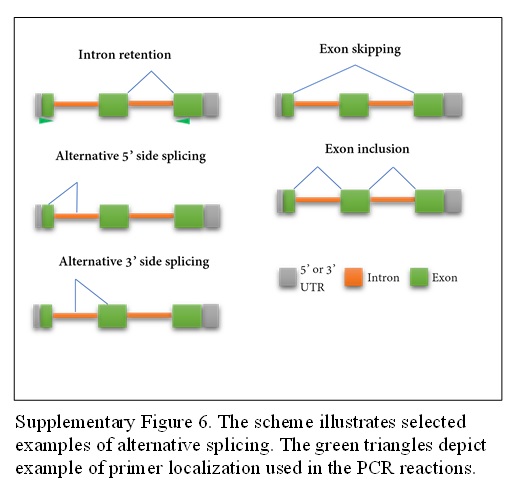

Supplement: Supplementary file 6 [file Image_6.jpg]

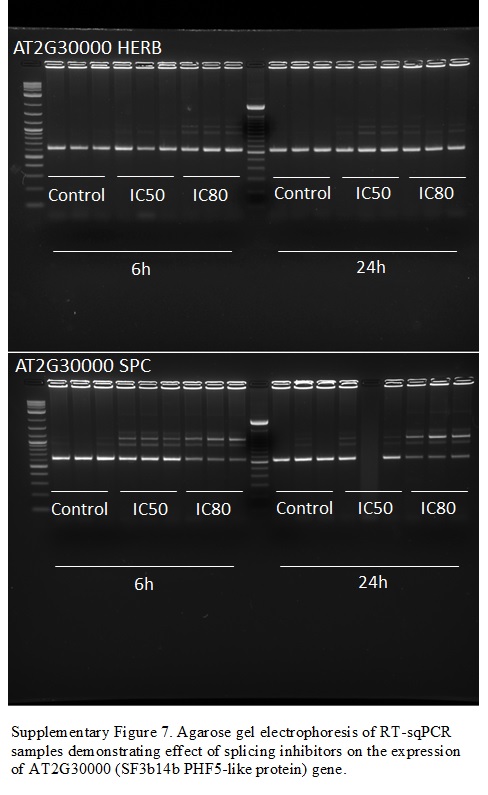

Supplement: Supplementary file 7 [file Image_7.jpg]

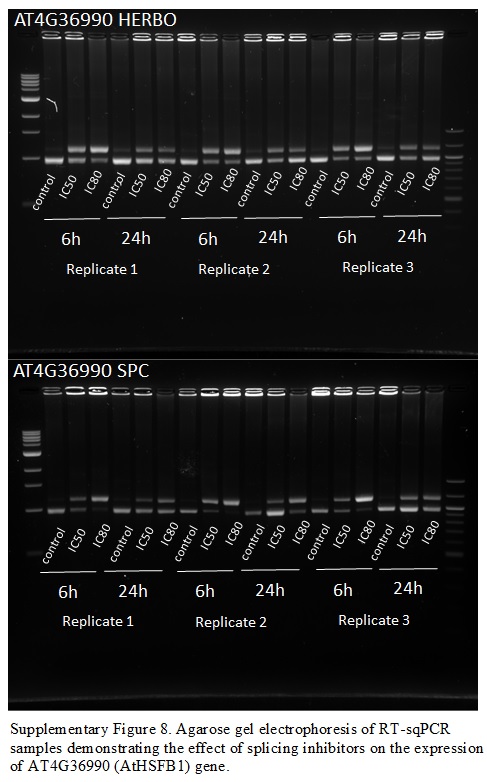

Supplement: Supplementary file 8 [file Image_8.jpg]

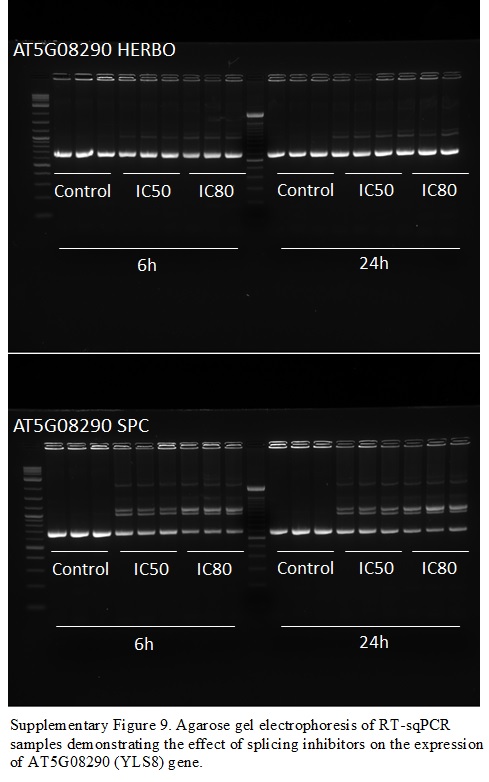

Supplement: Supplementary file 9 [file Image_9.jpg]

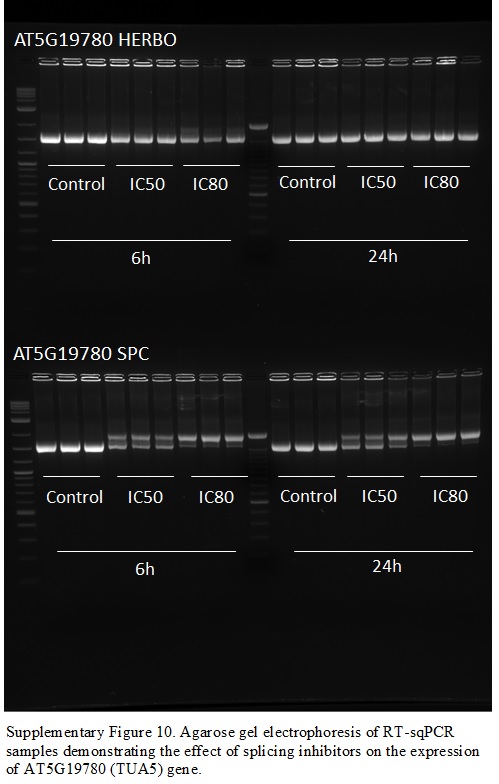

Supplement: Supplementary file 10 [file Image_10.jpg]

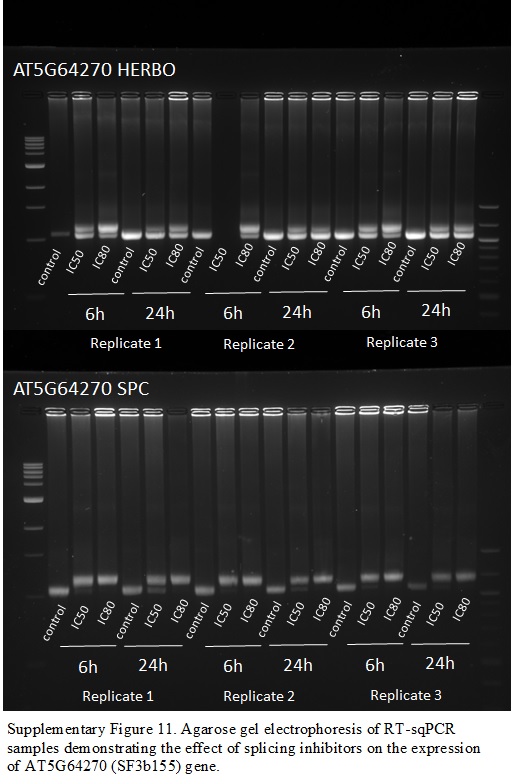

Supplement: Supplementary file 11 [file Image_11.jpg]
