# Supplementary material for: Spliceostatin C, a component of a microbial bioherbicide, is a potent phytotoxin that inhibits the spliceosome
Source: Front Plant Sci. 2023 Jan 12;13:1019938. doi: 10.3389/fpls.2022.1019938 (PMC9878571; doi:10.3389/fpls.2022.1019938)
Supplement: Supplementary file 12 [file Table_1.docx]

Supplementary Table 1. Sequences of primers used in RT-sqPCR experiment

| **Name** | **Sequence** |
| --- | --- |
| AT2G30000F | TGCTCTTTTTGCTTCGTCGT |
| AT2G30000R | TCGAGAGAAACATGTGTAACTTAAGA |
| AT2G23290F | AACAAAAATCGTTTCGTTCTTTTCC |
| AT2G23290R | TCTCAAATCATGAAATTGGCTCTT |
| AT2G40970F | TCGAGAGAAATGGAATCAGCCA |
| AT2G40970R | ACAAGCTACTGTGTTGTAGTGA |
| AT5G15710F | CGTGTTACTAAAAAGGACGAAGCT |
| AT5G15710R | CGAGTGAATGCTAATTTACAGAAAGC |
| AT1G18480F | ATCACGGAAAATGTCAAGCAGAG |
| AT1G18480R | AGAGACCTTGAACAATTATATTGAATAAGAA |
| AT1G77080F | GGCATAACCCTTATCGGAGAT |
| AT1G77080R | AGGAGGTACAACACTGATCCA |
| AT2G46830F | TCCATTTCCGTAGCTTCTGGT |
| AT2G46830R | ACAGAGTCAAATGTTACAGGAAGAC |
| AT5G45250F | CCACTGTGGAAGACAAGCCA |
| AT5G45250R | ACCGTGTGCATGATCACTGA |
| AT2G28390F | TGCCATTAGAGGAGACCAACC |
| AT2G28390R | TGCTCACTTTTCAAATAGTTTCAATTT |
| AT4G26410F | AAAACCAACTTCTAATTTGGAATCAAA |
| AT4G26410R | CCAAACCAAGCACACACATT |
| AT4G34270F | TGGTTGGATAGTCCGGTTGG |
| AT4G34270R | TGCATACGAGTCACTGCGTT |
| AT4G36990F | TTCTTACAAAGGTAGGACCAACA |
| AT4G36990R | CGTAAAAACACGGAAACCACCT |
| AT1G13440F | ATGGCGCCAAAGAAGGTTCTTC |
| AT1G13440R | CTACATCTTGGCCGCCACGATC |
| AT5G08290F | AAGGTTGAATCGAAACAGATCG |
| AT5G08290R | ACATAAACCCAACGGCTTCCA |
| AT5G09810F | AGAGTGAGAAAAATCGTAGAGCT |
| AT5G09810R | AGCTGCATTGTCACCCGATA |
| AT5G19780F | AGATCTCACTCTCACCGCCT |
| AT5G19780R | ATCTTCCCCAATGGTCGCTC |
| AT5G46630F | TCTTCCTTTGCTCCGTCGTC |
| AT5G46630R | TCGACAAAACAAACATAAAACAAGGT |
| AT5G64270F | CTGGCTTCATCTGGATAAACCCT |
| AT5G64270R | CAGGAGTGTAAGCTGCACCA |
| AT3G18780F | TGTCTCGTTGTCCTCCTCAC |
| AT3G18780R | AGGCATCAATTCGATCACTCAGA |
