# Supplementary material for: Spliceostatin C, a component of a microbial bioherbicide, is a potent phytotoxin that inhibits the spliceosome
Source: Front Plant Sci. 2023 Jan 12;13:1019938. doi: 10.3389/fpls.2022.1019938 (PMC9878571; doi:10.3389/fpls.2022.1019938)
Supplement: Supplementary file 13 [file Table_2.docx]

| **Gene** | **Accession No.** | **Primers** | **Sequence** | **Length** |
| --- | --- | --- | --- | --- |
| RALF1 | AT1G02900 | RALF1F | CGGAGGAAGAAGAAATGGACTCA | 93 |
|  |  | RALF1R | GCACACTGTTCCGTTTCAAAGA |  |
| RALF23 | AT3G16570 | RALF23F | TTCATAACTGGTCCGTCGCC | 80 |
|  |  | RALF23R | GCGGCACTCTGTTTCGAATG |  |
| RALF32 | AT4G14010 | RALF32F | CGTATGGAGCTTTGAGGAGGAA | 98 |
|  |  | RALF32R | ATAAGGGTTAGACGGTGGAGGA |  |
| RALF34 | AT5G67070 | RALF34F | CGGCGAATAGAGTGCCAT | 88 |
|  |  | RALF34R | TACGGATGAACGGGACCTC |  |
| UBQ10 | AT4G05320 | UBQ10F | ACTCGACTCTGTTTAGTGCAGTT | 90 |
|  |  | UBQ10R | ACAGCTCAACACTTTCGCTACA |  |
| UBC | AT5G25760 | UBCF | GGTACAGCGAGAGAAAGTAGCA | 89 |
|  |  | UBCR | GTCCTTTGATAAGAGCGGTCCA |  |

Supplementary Table 2. Primers used in quantitative real-time PCR (RT-qPCR) analysis.
