# Supplementary material for: Spliceostatin C, a component of a microbial bioherbicide, is a potent phytotoxin that inhibits the spliceosome
Source: Front Plant Sci. 2023 Jan 12;13:1019938. doi: 10.3389/fpls.2022.1019938 (PMC9878571; doi:10.3389/fpls.2022.1019938)
Supplement: Supplementary file 14 [file Table_3.docx]

| **#** | **Name** | **GO** | **Number of introns** | **Category** |
| --- | --- | --- | --- | --- |
| **1** | AT2G28390 | MONENSIN SENSITIVITY1 (MON1) | 13 | Stable expression/constitutive splicing |
| **2** | AT4G26410 | RGS1-HXK1 INTERACTING PROTEIN 1 (RHIP1) | 7 |  |
| **3** | AT4G34270 | TAP42 INTERACTING PROTEIN OF 41 KDA (TIP41) | 6 |  |
| **4** | AT1G13440 | Glyceraldehyde-3-phosphate dehydrogenase (GAPDH) | 3 |  |
| **5** | AT5G08290 | YELLOW-LEAF-SPECIFIC GENE 8 (YLS8) | 1 |  |
| **6** | AT5G09810 | Actin 7 (ACT7) | 4 |  |
| **7** | AT3G18780 | Actin 2 (ACT2) | 3 |  |
| **8** | AT5G19780 | Tubulin alpha 5 (TUA5) | 4 |  |
| **9** | AT5G46630 | ADAPTOR PROTEIN-2 MU-ADAPTIN (AP2M) | 10 |  |
| **10** | AT4G36990 | Heat shock factor 4 (ATHSFB1) | 1 |  |
| **11** | AT1G77080 | FLOWERING LOCUS M (FLM) | 5 | Regulation factors/alternative splicing |
| **12** | AT2G46830 | Circadian clock associated 1( CCA1) | 7 |  |
| **13** | AT5G45250 | Disease resistance protein family( RPS4) | 4 |  |
| **14** | AT5G64270 | SF3b155 | 2 | Splicing factors |
| **15** | AT2G30000 | SF3b14b PHF5-like protein | 1 |  |
| **16** | AT2G23290 | MYB DOMAIN PROTEIN 70 (ATMYB70) | 0 | Intronless |
| **17** | AT2G40970 | 1R-MYB PROTEIN (ATMYBC1) | 0 |  |
| **18** | AT5G15710 | Galactose oxidase/kelch repeat superfamily protein | 0 |  |
| **19** | AT1G18480 | SHEWENELLA-LIKE PROTEIN PHOSPHATASE 2(ATSLP2) | 0 |  |

Supplementary Table 3. Arabidopsis genes selected for reverse transcriptase semi-quantitative PCR assays.
