# Supplementary material for: Spliceostatin C, a component of a microbial bioherbicide, is a potent phytotoxin that inhibits the spliceosome
Source: Front Plant Sci. 2023 Jan 12;13:1019938. doi: 10.3389/fpls.2022.1019938 (PMC9878571; doi:10.3389/fpls.2022.1019938)
Supplement: Supplementary file 16 [file Table_5.docx]

| **Mutation** | **Binding free-energy (kcal/mol)** |
| --- | --- |
| WT (Y36) | -63.12 |
| Y36L | -60.14 |
| Y36W | -65.44 |
| Y36A | -53.01 |
| Y36C | -55.46 |
| Y36R | -59.18 |
| Y36E | -44.78 |

Supplementary Table 5. The binding free-energies data of wild-type Y36 and mutated homology model of SF3B1-PHF5A.
