# Supplementary material for: Spliceostatin C, a component of a microbial bioherbicide, is a potent phytotoxin that inhibits the spliceosome
Source: Front Plant Sci. 2023 Jan 12;13:1019938. doi: 10.3389/fpls.2022.1019938 (PMC9878571; doi:10.3389/fpls.2022.1019938)
Supplement: Supplementary file 17 [file DataSheet_1.docx]

> AT5G64270_4.6f

GATTTTCTCGCTCTACTCCCACAATCATCCGCAGACGAGTTCCGATTACCGGTGAGTTATTCTCCAATCTTCGTTTCGATATACGATTCTATTCTCTCGTATTTGATTGATTCCGATTGTTTTCAATTTCGTTTTGTTTTCTTCTGATTCACTGCTTTGTTGATTTTCTCAGTTCTTTCTTTTTTTGCTGGAGAAAGCTTCGTTGAGTATCTGATAATGGCGGATCTAGATCCAGAGATCGCTAAAACTCAGGAGGAAAGACGGAAGATGGAAGCAGACCTTGCTTCTCTCACTTCTCTGACTTTCGATCGCGATCTATACGGAGGTAACGACCGTGCTTCCTACTCGACTTCTATTGCACCTAACGAGGAGGATGATGCGAATCTGGACACCACTGGTTCTCTTGTGGCTCAGCGCCTCGCGTCTTATACTGCTCCTAGGTCAATTCTTAACGACGTGGCTCGTCCTCACAACGAAGATGACGATGTGGGATTTAAACCTAGGCAGAGTATTGCCGAACGTGAGGGTGAATATAGGAATAGGAGACTCAATCGGGTTCTTTCTCCGGATAGAGTTGATGCCTTTGCTATGGGGCGACAAAACGCCGGATGCGAGTGTTCGTACTTTACACGGACCATATGAGGGAGACGGCTTTTGCAGAGGGAGAAAGAGGAAACTATGAGGCTCATTGCCAAGAAGAAGAAAGAGGAAGAAGAAGCTGCTGCGAAGCATCAGAAAGATTCTGCTCCTCCTCCTCCTGCTTCCTCTTCTTCTTCCTCTTCCAAGAGGAGACACAGGTGGGATCTCCCTGAAGAAAGGCGGTGCTGCTGCAAAGAAAGGCTAAAGCAGCAAGTTCAGATTGGGATTTACCTGATGCAGCTCCAGGGATTGGACGATGGGATGCTCCTACTCCAGGGAGAGTTTCTGATGCCACACCATCTGCTGGGACGAAGGAACAGTGGGACGAAACTCCTACACCTGGTCGTGTGACTGATTCTGATGCAACACCTGGTGTGGTGTTACTCCCGGTGCTACTCCTTTCAGTGTACTTGGGACGGCTTGCACGCTACCCAAGCGTCACGTCAGTTGGACGAACACAGCACTATGGGGAGTGCTACACTATGATGAGTCACTCCTTGATGCAGCTACACTCCTGCTGAATAATATAATAGACGTCAGATTTCTCGAGCGCGCATGTCGCTCTTCCTAATGAGTCGCTATAATTCCGCGAAACCAATGGTGATTTCCA

>AT5G64270_4.6r

ATTTGGTTTAAGAGATATTCTGCGTCTCACCCTCTGCCAGAGAAGGGTGAACGCCAATTAAGGTGACACCTATAGAAAGTGGTGTATTCGCTCGAGGGATCCGAAATTCAGGAGGTAAACCATGATCTGGCTTCATCTGGATAAACCTAGTTCATCCTTTTTCTCACGCTCTACCTCCCCCACATCATCGCAGACGAGTTCCGATTACCCGGTGAGTTATTTCTCCAATCTTCGTTTCGATATACGATTCTATTCTTCTCGTATTTGATTGATTCCGATTGTTTTCAATTTCGTTTTGTTTTCTTCTGATTCACTGCTTTGTTGATTTTTCTCAGTTCTTTCTTTTTTTGCTGGAGAAAGCTTCGTTGAGTATCTGATAATGGCGGATCTAGATCCAGAGATCGCTAAAACTCAGGAGGAAAGACGGAAGATGGAAGCAGACCTTGCTTCTCTCACTTCTCTGACTTTCGATCGCGATCTATACGGAGGTAACGACCGTGCTTCCTACTCGACTTCTATTGCACCTAACGAGGAGGATGATGCGAATCTGGACACCACTGGTTCTCTTGTGGCTCAGCGCCTCGCGTCTTATACTGCTCCTAGGTCAATTCTTAACGACGTGGCTCGTCCTCACAACGAAGATGACGATGTGGGATTTAAACCTAGGCAGAGTATTGCCGAACGTGAGGGTGAATATAGGAATAGGAGACTCAATCGGGTTCTTTCTCCGGATAGAGTTGATGCCTTTGCTATGGGCGACAAAACGCCGGATGCGAGTGTTCGTACTTACACGGACCATATGAGGGAGACGGCTTTGCAGAGGGAGAAAGAGGAAACTATGAGGCTCATTGCCAAGAAGAAGAAAGAGGAAGAAGAAGCTGCTGCGAAGCATCAGAAAGATTCTGCTCCTCCTCCTCCTGCTTCCTCTTCTTCTTCCTCTTCCAAGAGGAGACACAGGTGGGATCTCCCTGAAGAAGGCGGTGCTGCTGCAAAGAAGGCTAAAGCAGCAAGTTCAGATTGGGATTTACCTGATGCAGCTCCAGGGATTGGACGATGGGATGCTCCTACTCCAGGGAGAGTTTCTGATGCCACACCATCTGCTGGACGAAGGAACAGGTGGGACGAAACTCCTACACCTGGTCGTGTGACTGATTCTGATGCAACACCTGGTGGTGGTGTTACTCCCGGTGCTACTCCTTCAGGTGTTACTTGGGACGGGCTTGCCACGCCTACCCCAAAGCGTCAACGTTCAAGGTGGGACGAAACACCAGCCACTATGGGAGTGCTACAGCTATGTC

>AT5G64270_6.2f

CTTTTCTACGCTCTACTCCCACAATCATCCGCAGACGAGTTCCGATTACCGGTGAGTTATTCTCCAATCTTCGTTTCGATATACGATTCTATTCTCTCGTATTTGATTGATTCCGATTGTTTTCAATTTCGTTTTGTTTTCTTCTGATTCACTGCTTTGTTGATTTTCTCAGTTCTTTCTTTTTTTGCTGGAGAAAGCTTCGTTGAGTATCTGATAATGGCGGATCTAGATCCAGAGATCGCTAAAACTCAGGAGGAAAGACGGAAGATGGAAGCAGACCTTGCTTCTCTCACTTCTCTGACTTTCGATCGCGATCTATACGGAGGTAACGACCGTGCTTCCTACTCGACTTCTATTGCACCTAACGAGGAGGATGATGCGAATCTGGACACCACTGGTTCTCTTGTGGCTCAGCGCCTCGCGTCTTATACTGCTCCTAGGTCAATTCTTAACGACGTGGCTCGTCCTCACAACGAAGATGACGATGTGGGATTTAAACCTAGGCAGAGTATTGCCGAACGTGAGGGTGAATATAGGAATAGGAGACTCAATCGGGTTCTTTCTCCGGATAGAGTTGATGCCTTTGCTATGGGCGACAAAACGCCGGATGCGAGTGTTCGTACTTACACGGACCATATGAGGGAGACGGCTTTGCAGAGGGAGAAAGAGGAAACTATGAGACTCATTGCCAAGAAGAAGAAAGAGGAAGAAGAAGCTGCTGCGAAGCATCAGAAAGATTCTGCTCCTCCTCCTCCTGCTTCCTCTTCTTCTTCCTCTTCCAAGAGGAGACACAGGTGGGATCTCCCTGAAGAAGACGGTGCTGCTGCAAAGAAGGCTAAAGCAGCAAGTTCAGATTGGGATTACCTGATGCAGCTCCAGGGATTGGACGATGGGATGCTCCTACTCCAGGGAGAGTTTCTGATGCCACACCATCTGCTGGACGAAGGAACAGGTGGGACGAAACTCCTACACCTGGTCGTGTGACTGATTCTGATGCAACACCTGGTGGTGGTGTTACTCCCGGTGCTACTCCTTTCAGGTGTTACTTTGGACGGCTTGCCACGCCTACCCCAAAGCGTCACGTTCAAGGTGGGACGAAACACCAGCCACTATGGGGGAGTGCTACACCTATGGGTGGAGTCCACCTCCTGATGCAGCTTAACACCTCCTGTCAGATCTCGAGCCGGCCGCATGTGCGATCTCCTTATAGTGGAGTCGTAATAGTTTCGCGGGCGAACCCTATGCTTAATTTCTGA

>AT5G64270_6.2r

GATCAAAGCATTACATTCAACGAATCGACGACTGTACGTAATCGTTCACTGCAAGACGGTTTAACGCATTTAGTGGACCTATAGAAGTGTGTATCGCTCGAGGGATCCGGAATTCAGAGTTAAAACATGATCTGCTTCATCTGATAAAGCCCTAGTCATCGTTTTCTCACGCTCTACCTCCCACAATCATCGCAAGACGAGTTCCCGATTACCGGTGAGTTATTCTCCAATTCTTCGTTTCGATAATACGATTCTATTCTCTCGTAATCTGATTGATTTCCGATTGTTTTCAAATTCGTTTTTGTTTTTCTTCTGATTCACTGCTTGTTGATTTTCTCAGTTCTTTCTTTTTTTGCTGGAGAAAGCTTCGTTGAGTATCTGATAATGGCGGATCTAGATCCAGAGATCGCTAAAACTCAGGAGGAAAGACGGAAGATGGAAGCAGACCTTGCTTCTCTCACTTCTCTGACTTTCGATCGCGATCTATACGGAGGTAACGACCGTGCTTCCTACTCGACTTCTATTGCACCTAACGAGGAGGATGATGCGAATCTGGACACCACTGGTTCTCTTGTGGCTCAGCGCCTCGCGTCTTATACTGCTCCTAGGTCAATTCTTAACGACGTGGCTCGTCCTCACAACGAAGATGACGATGTGGGATTTAAACCTAGGCAGAGTATTGCCGAACGTGAGGGTGAATATAGGAATAGGAGACTCAATCGGGTTCTTTCTCCGGATAGAGTTGATGCCTTTGCTATGGGCGACAAAACGCCGGATGCGAGTGTTCGTACTTACACGGACCATATGAGGGAGACGGCTTTGCAGAGGGAGAAAGAGGAAACTATGAGACTCATTGCCAAGAAGAAGAAAGAGGAAGAAGAAGCTGCTGCGAAGCATCAGAAAGATTCTGCTCCTCCTCCTCCTGCTTCCTCTTCTTCTTCCTCTTCCAAGAGGAGACACAGGTGGGATCTCCCTGAAGAAGACGGTGCTGCTGCAAAGAAGGCTAAAGCAGCAAGTTCAGATTGGGATTACCTGATGCAGCTCCAGGGATTGGACGATGGGATGCTCCTACTCCAGGGAGAGTTTCTGATGCCACACCATCTGCTGGACGAAGGAACAGGTGGGACGAAACTCCTACACCTGGTCGTGTGACTGATTCTGATGCAACACCTGGTGGTGGTGTTACTCCCGGTGCTACTCCTTCAGGTGTTACTTGGGACGGGCTTGCCACGCCTACCCCAAAGCGTCAACGTTCAAGGTGGGACGAAACACCAGCCACTATGGGGGAGTGCTACACCCAGGGGGT

>AT5G19780_16.6r

GAAGAAATGGTCTCTGTTGCTAGAGCGTTTGTCTGTAGATTACGGAAGAAGTCTAAGCTTGGTTTTACCATATACCCTTCTCCTCAGGTACTCTTTCTTTTGCTAGACACTGAAGTAGAACCTCTTACATGACATGTTTCTATAGTGGTCTAATTTGGATGAAACTTGGTTTTAATGCATATTCCTCTGTGATGTCAGGTTTCTACTGCTGTTGTAGAGCCTTACAACAGTGTGCTTTCAACGCATTCCCTTCTTGAACATACCGATGTAGCTGTCCTCTTGGATAACGAAGCCATCTATGACATTTGCCGCAGATCCCTAGATATCGAGAGGCCAACCTACACAAACTTGAACAGGTTGATATCACAGATCATTTCATCCTTGACAACATCTTTGAGGTTTGATGGTGCCATCAACGTGGATATCACTGAGTTCCAGACCAATCTTGTCCCATATCCCCGTATCCATTTCATGCTGTCATCTTATGCACCAGTCATCTCAGCCGCCAAGGCTTACCACGAGCAGCTATCAGTCCCTGAGATCACCAATGCCGTGTTTGAGCCAGCGAGCATGATGGCAAAGTGTGACCCAAGGCACGGAAAGTACATGGCATGTTGTTTGATGTACCGAGGAGATGTTGTTCCCAAAGATGTTAATGCTGCCGTTGGCACCATCAGACAAAGAGGACTGTTCAGGTTTGTTGACTGGTATGATCTAGAAATGGAAATATAGCTGCTAATTTTTCTACCTTTATTGTTGTTATTAATAATAGTACCTCCTTTGGCCATGGGTTTTTGTTTTTGCAAGGGTGCCAACTGGATTCAAATGGGTGAAATCAACTACAACCCTCAAACAGTTGTTTCCAGGTGGGTGACCTTCCGCTGAGGTTCAGAAAGAGCTTGTTATGCATGATCCAGTTAACCAACCCAGCAGTTGGCAAGTGTTCCTCACGATTCCGACCACAGTTGGATCTCCATGTATGGCCAAAGGCCAATTTCTGGACTTGTTACGTTGTGTCAGGAATGGAGCAGTGTGTAATTCTCTTGAGGGCACTGTCGAAACTTGGCGCACTGGTAAAGGACATCCAGAGTGGGCCTGTACGATGT

>AT5G19780_18.6b

GGGGTGGGGACTTACTGTCTCGACATGGATCCAGCCCGACGGAATGATGCCGAGGTATAAACCCTAATTCTCTCTTTTATACTCTGTTTCTGTGGATCTGAAGGTTTTTGAGAGACGAATGTTTTGCTCTGTTGCAGTGATACTACAGTTGGTGTTGCACACGATGCGTTCAATACTTTCCTTAGCGAGACTGGAGCTGGGAAGCATGTTCCTAGGGCTGTCTTCGTTGATCTCGAGCCTACCGTTATCGACGAAGTTCGTACTGGTACTTACCGTCAACTTTTCCATCCAGAGCAGCTCATTTCTGGGAAAGAAGATGCTGCTAACAACTTCGCTAGAGGACATTACACTGGTGAGAATCCACTTTCTGCTTGTTCTAACTTGTTAGGGTTTTTGAAATGGTCCTTAGAATGCTTCTTGTAATTGCAGTTGGAAAGGAAATTGTGGATCTATGTCTTGACCGTGTGAGGAAGCTTGCCGACAACTGTACTGGCTTACAAGGGTTTTTGGTGTTCAATGCTGTTGGTGGTGGAACTGGTTCTGGATTGGGTTCTCTGTTGCTAGAGCGTTTGTCTGTAGATTACGGAAAGAAGTCTAAGCTTGGTTTTACCATATACCCTTCTCCTCAGGTACTCTTTCTTTTGCTAGACACTGAAGTAGAACCCTCTTACATGACATGTTTCTATAGTGGTCTAATTTGGATGAAACTTGGTTTTAATGCATATTCCTCTGTGATGTCAGGTTTCTACTGCTGTTGTGAGCCTTACACAGGTGTGTGCTTTCAACCGCATTTCCCTTTCTTGAACATACCGATTGTAGCTGGTCCCTCTCTTTGAATAAACCGAAGCCATTTTATTGAACATTGCCCGCAAGAATCCTAAGAATATTCGAGGAGGCCCACCCTTAACCACAACTTGACCAGGTTGATATTCCACGAATCATTCAATCCCATGACAACCTTCTTTGGGTTGATGGGCCACTCCACCGAGGAATTTCACTGGAATCTGAACACCTTGTCC

>AT5G19780_18.6b

GGCGGGATGGTCTCTGTTGCTAGAGCGTTTGTCTGTAGATTACGGAAGAAGTCTAAGCTTGGTTTTACCATATACCCTTCTCCTCAGGTACTCTTTCTTTTGCTAGACACTGAAGTAGAACCTCTTACATGACATGTTTCTATAGTGGTCTAATTTGGATGAAACTTGGTTTTAATGCATATTCCTCTGTGATGTCAGGTTTCTACTGCTGTTGTGGAGCCTTACAACAGTGTGCTTTCAACGCATTCCCTTCTTGAACATACCGATGTAGCTGTCCTCTTGGATAACGAAGCCATCTATGACATTTGCCGCAGATCCCTAGATATCGAGAGGCCAACCTACACAAACTTGAACAGGTTGATATCACAGATCATTTCATCCTTGACAACATCTTTGGGGTTTGATGGTGCCATCAACGAGGATATCACTGAGTTCCAGACCAATCTTGTCCCATATCCCCGTATCCATTTCATGCTGTCATCTTATGCACCAGTCATCTCAGCCGCCAAGGCTTACCACGAGCAGCTATCAGTCCCTGAGATCACCAATGCCGTGTTTGAGCCAGCGAGCATGATGGCAAAGTGTGACCCAAGGCACGGAAAGTACATGGCATGTTGTTTGATGTACCGAGGAGATGTTGTTCCCAAAGATGTTAATGCTGCCGTTGGCACCATCAAGACAAGAGGACTGTTCAGTTTGTTGACTGGTATGATCTAGAAATGGATATAGCTGCTAATTTTTTCTACCTTTATGTGTTATTAATAATAGTAACCTCCTTTGGCATGGGTTTGTTTTGCAAGGTGCCAACTGGGATTCAATGGTGGAATCAACTACACCTCCACAGTTGTTCCAAGGTGGGTGACCCTCCGCTAAGGGTTCAAGAGAGAGCTTGTATGCATGATCAGTAACAAACACAGCAGTCCGCAAGGTGGTTTCTCTCACGGACGAACCACAAGTTGATTCTCATGCATTGCCAAAGAGGCCAATCTGTGCACTGTTACGGTGGGTGAAGTAATGGAGGAAGGTATTCTCTGGAGTCACTTGAAGACTGACCGCCACTGAAAGACTTCCAAATGAGTCTAATGTGACCGATTGAAGACTTTGA

>AT5G19780_16.6

TTCGGGGGACTTACTGTCTCGACATGGATCCAGCCCGACGGAATGATGCCGAGGTATAAACCCTAATTCTCTCTTTTATACTCTGTTTCTGTGGATCTGAAGGTTTTTGAGAGACGAATGTTTTGCTCTGTTGCAGTGATACTACAGTTGGTGTTGCACACGATGCGTTCAATACTTTCTTTAGCGAGACTGGAGCTGGGAAGCATGTTCCTAGGGCTGTCTTCGTTGATCTCGAGCCTACCGTTATCGACGAAGTTCGTACTGGTACTTACCGTCAACTTTTCCATCCAGAGCAGCTCATTTCTGGGAAAGAAGATGCTGCTAACAACTTCGCTAGAGGACATTACACTGGTGAGAATCCACTTTCTGCTTGTTCTAACTTGTTAGGGTTTTTGAAATGGTCCTTAGAATGCTTCTTGTAATTGCAGTTGGAAAGGAAATTGTGGATCTATGTCTTGACCGTGTGAGGAAGCTTGCCGACAACTGTACTGGCTTACAAGGGTTTTTTGGTGTTCAATGCTGTTGGTGGTGGAACTGGTTCTGGATTGGGTTCTCTGTTGCTAGAGCGTTTGTCTGTAGATTACGGAAAGAAGTCTAAGCTTGGTTTTACCATATACCCTTTCTCCTCAGGTACTCTTTCTTTTGCTAGACACTGAAGTAGAACCTCTTTACATGACATGTTCTATAGTGTCTAATTTGGATGAAACTTGTTTTTAATGCATATTCCTTCTGGTGATGTCAGGTTTCTACTGCTGTTGTAAGAGCCTTACAAACAGGTGGTGCTTTCACGCATTCCCTTCGTGAAACAATACCGATGTAGCTGTCCCTCCTGCAATAACGAGTCATCATTGAACTTGCCGCAGAATCCCTAAGAATATTCGAGAAGGACTAAGCTACCCAACTGAACAGGTTGGATTGCAGATTCATCAATCTTGACAACACTA

>AT5G08290_11.7er

TAAATGCAAGCCGGTGCGAGTCAGCATAAGACGATGCCAGAATGTTTAGACAACCGTAAGGCCATAAGCTAATTCCAGGAAGTCAAGCGTTAGCGATTGAATTCACCGGAGATGGCTGCCCGCTGCGGCCATAAAAAATATTACACACTCCAGCGCTGATGAATCCCTATGATTTTGGTAAAAATCATTAAGTAAGGTGGACACACATCTTGTCATATGATTAAATGGTTTCGCGAAAAAATCAATAATCAGACAACAAGATGTGCGAACTCGATATTTTTACACGACTCTCTTTACCAATTCTGCCCCGAATTACACTTAAAACGACTCAACAGCTTAACGTTGGCTTGCCACGCCATTACTTGACTGTAAAACTCTCACTCTTACCGAACTTGGGCCGTAACCTGCCAACCCAAAGCGAGAACAAAACATAACATCAAACGAATCGACCGATTGTTAGGTAATCGTCACCTGCAGGAAGGTTTAAACGCATTTAGGTGACACTATAGAAGTGTGTATCGCTCGAGGGATCCGAATTCAGGAGGTAAAAACCATGATAAGGTTGAATCGAAACAGATCGGAAAATCGTCGAGAGAGAGAGAGAGAGAGAATGTCGTATCTTCTTCCACATCTGCACCCCGGTTGGGCTGTTGATCAGTCGATTCTGGCCGAGGAAGAGCGTCTCGTCGTCATTCGTTTCGGCCATGACTGGGATGAGACCTGTATGCAGGTCTGTCAAACGTTTCTTCCTCTCTTATCTTTATATTTCGATCTTGAGCTTGATTATATGTTTTTGATTCGATTTGTAGTTCATAGCTCTCGATTTGGATCCTTTCGTACTGGAAAATTGCGATACTTTGTGGTGATTGTTTGAAAATTGTGTTCCTTAGCTCAGAACTGATGAAATTAGGGTTTCACTCAAAAAAATCCTAGTTGTTGGAGTTGGTTACTATGTAGAAGGTTTTGAATGTGTCCGTTTTAGTGTCCATTTCTGTGATTTATGCTTCGTTTTGTTCTTGCGAATTTGGTGATCTTGGAATATTCCGGATATGTTGTGTTGATTGTTTGAAACTTTAGCCATTCGTGTGATCTTTATTATCTTGGAACTGATTAAAATTTTAGGGCTTCACTTACAATTGATTCTAGTATTCAGTTTCTGGTTTCTGTTTTGATTCGAAGTTTTTGATTTTTGTAGATGGATGAGGTGCTTGCGTCTGTTGCTGAGACGATTAAGAACTTGCAGTCATTATCTGGGACCTCACCTTCC

>AT5G08290_10.1df

CTCCAGGGGGGAGAGAGAGAGAGAGATGTCGTATCTTCTTCCACATCTGCACTCCGGTTGGGCTGTTGATCAGTCGATTCTGGCCGAGGAAGAGCGTCTCGTCGTCATTCGTTTCGGCCATGACTGGGATGAGACCTGTATGCAGGTCTGTCAAACGTTTCTTCCTCTCTTATCTTTATATTTCGATCTTGAGCTTGATTATATGTTTTTGATTCGATTTGTAGTTCATAGCTCTCGATTTGGATCCTTTCGTACTGGAAAATTGCGATACTTTGTGGTGATTGTTTGAAAATTGTGTTCCTTAGCTCAGAACTGATGAAATTAGGGTTTCACTCAAAAAAATCCTAGTTGTTGGAGTTGGTTACTATGTAGAAGGTTTTGAATGTGTCCGTTTTAGTGTCCATTTCTGTGATTTATGCTTCGTTTTGTTCTTGCGAATTTGGTGATCTTGGAATATTCCGGATATGTTGTGTTGATTGTTTGAAACTTTAGCCATTCGTGTGATCTTTATTATCTTGGAACTGATTAAAATTTTAGGGCTTCACTTACAATTGATTCTAGTATTCAGTTTCTGGTTTCTGTTTTGATTCGAAGTTTTTGATTTTTGTAGATGGATGAGGTGCTTGCGTCTGTTGCTGAGACGATTAAGAACTTTGCAGTCATTTATCTGGTGGACATCACTGAGGTTCCAGACTTCAACACCATGTACGAGCTGTACGATCCTTCTACGGTCATGTTCTTCTTCAGGAACAAGCACATCATGATCGATCTTGGAAACTGGTAACAACAACAAGATCAACTGGGCTCTCAAGGGACAAGCAGGAGTTCATTGTTATCATTGAGACTGTCTACCGTGGTGCAAGGAAAGGGTCGTGGTTTGGTGATTGCTTCCAAAAAGATTACTCCACCAAATACCGTTACTAATCGAGCTTTCCCAACACTATCTAGTTTGTTAAACCCATTGAGTCTAGTGATTCTGGTCAGCTGAAATATCCCGTGAGCTGCTTTCATTCATAATATGCTTGATGATGATGTGATCTGGCTTTTGCTACTGTTCCGTGTCTCATTTATGTATGTACCTACCTGCCTCGAACATGATCCAGTGAAATTGAAATTTGAGCCGTGGTTATTGTATCATGTTCACTCTGATTCGATCCTCGGAGCATACACGTCTATAGGTTACCTAAGTGCATTACTCTGAC

>AT5G08290_10.1dr

CTGGCATAATGTCCAACGTCATGCATCGTAGATGCTTCCCTGGACTAGTGAGTAATCCAAGCCAAGGTCATTCCGAGGAATAGGTATGCGGCGATCGAGTGCTCTGCCGCGTCCAATACGGGATAATACGCGCACATAGCAGAACTTAAAAGTGCTCATCATGAAAACGTTCTTCGGGCGAAAACTCTCCAAGGGATCTTACCCGCTGTTGAAGAATCCAGTCGATGTAAACCCCACCTCGTGCACCCCAACTGATCTTCAGCATCTTTTTACTTTCACCAGCGTTCTGGGTGAGCAAAAACAGGAAGGCAAAATGCCGCAAAAAAGGGAATAAGGGCGACACGGAAATGTTGAATACTCATACTCTTCCTTTTTCAATATTATTGAAGCATTTATCAGGGTTATTGTCTCATGAGCGGATACATATTTGAATGTATTTAGAAAAATAAACAAATAGGGGTTCCGCCCGCGAAATTAATACGACTCACTATAGGGAGACGCACATGCGGCCGCCTCGAGAATTCTGACGTCTTAATTAATTATTATCAGAAGGTTGAATCGAAACAGATCGGAAAATCGTCGAGAGAGAGAGAGAGAGAGAGAATGTCGTATCTTCTTCCACATCTGCACTCCGGTTGGGCTGTTGATCAGTCGATTCTGGCCGAGGAAGAGCGTCTCGTCGTCATTCGTTTCGGCCATGACTGGGATGAGACCTGTATGCAGGTCTGTCAAACGTTTCTTCCTCTCTTATCTTTATATTTCGATCTTGAGCTTGATTATATGTTTTTGATTCGATTTGTAGTTCATAGCTCTCGATTTGGATCCTTTCGTACTGGAAAATTGCGATACTTTGTGGTGATTGTTTGAAAATTGTGTTCCTTAGCTCAGAACTGATGAAATTAGGGTTTCACTCAAAAAAATCCTAGTTGTTGGAGTTGGTTACTATGTAGAAGGTTTTGAATGTGTCCGTTTTAGTGTCCATTTCTGTGATTTATGCTTCGTTTTGTTCTTGCGAATTTGGTGATCTTGGAATATTCCGGATATGTTGTGTTGATTGTTTGAAACTTTAGCCATTCGTGTGATCTTTATTATCTTGGAACTGATTAAAATTTTAGGGCTTCACTTACAATTGATTCTAGTATTCAGTTTCTGGTTTCTGTTTTGATTCGAAGTTTTTGATTTTTGTAGATGGATGAGGTGCTTGCGTCTGTTGCTGAGACGATTAAGAACTTTGCAGTCATTATCTGGGGAACACCCCCTTCC

>AT5G19780_18.6b

GGATCCTTCGCTCTCTGACTTAGCGAGGTCACCACCTGGAACAACTGTTGGAGGTTGGTAGTTGATTCCACATTTGAACCCAGTTGGGCACCTGCAAAACAAAACCCATGCCAAAGGAGTTACTATATAATAACAACATAAAGGTAGAAAAATTAGCAGCTATATCCATTTCTAGATCATACCAGTCAACAAACTGAACAGTCCTCTTTGTCTTGATGGTGCCAACGGCAGCATTAACATCTTTGGGAACAACATCTCCTCGGTACATCAAACAACATGCCATGTACTTTCCGTGCCTTGGGTCACACTTTGCCATCATGCTCGCTGGCTCAAACACGGCATTGGTGATCTCAGGGACTGATAGCTGCTCGTGGTAAGCCTTGGCGGCTGAGATGACTGGTGCATAAGATGACAGCATGAAATGGATACGGGGATATGGGACAAGATTGGTCTGGAACTCAGTGATATCCTCGTTGATGGCACCATCAAACCCCAAAGATGTTGTCAAGGATGAATGATCTGTGATATCAACCTGTTCAAGTTTGTGTAGGTTGGCCTCTCGATATCTAGGGATCTGCGGCAATGTCATAGATGGCTTCGTTTCCAAGAGGACAGCTACATCGGTATGTCAAGAGGGATGCGTTGAAGCACACTGTTGTAAGGTCACACAGCAGAAAACCTGACATCACAGAGGATATGCATTAAACCAGTTCATCCAATTAGACACTATAGAACATGTCATGTAAGAGGTCTACTTCGGGCTAGCAAAGGAAGATACCTGAGGAAGGGATATGTAAACAAGCTAAACTTCTTCGAATCACGACAACCTTCAGCACGAAGACCATCAGACAGTTCCCCCAACAGATGAACCCAAAACCTGGAGCATACGGTTCGGCAGTTCTACACGTAAATAGACACATTCTTTCATGCATTAAGAACTCCAAGCATTCAAACCTCCAGTAAAAGGGAGGGTTCCCAGGGAGGCCTTCGGATTTGCCATTCTTCAAATGGTTCCGAGGAGTGCGAGTCGACGATTTCTAACGAGGTGGATACGAAAGCTAGATGTTCCGTCAGTCCGTAGGAATTGACTCGTGCAACATGGATTCTGCAAGCACTTCTCCAACTCGACCGACATAAAGATAGTACGCATCGCGTGATCATCGAGAGTCAGATCATGACGTGCAAGCTATTCATCTCGACATCAGTCGCCAGAGACGACGAGGCGAATGTATATACGATTCGGCGGCTAGGTATTCGGGACTTTTCATATGGTCGAACTAGTTTAGAGAGATCTCGCTCTGTTCTGCAGAGATTGGCAGTCGAGTCATGATATGTCGAGCCTAGTCATCGAGCAATCAGCTCTGACAGA

>AT5G08290_11.7ef

CATCGAAGAAGAGAGAGAGAGATGTCGTATCTTCTTCCACATCTGCACCCCGGTTGGGCTGTTGATCAGTCGATTCTGGCCGAGGAAGAGCGTCTCGTCGTCATTCGTTTCGGCCATGACTGGGATGAGACCTGTATGCAGGTCTGTCAAACGTTTCTTCCTCTCTTATCTTTATATTTCGATCTTGAGCTTGATTATATGTTTTTGATTCGATTTGTAGTTCATAGCTCTCGATTTGGATCCTTTCGTACTGGAAAATTGCGATACTTTGTGGTGATTGTTTGAAAATTGTGTTCCTTAGCTCAGAACTGATGAAATTAGGGTTTCACTCAAAAAAATCCTAGTTGTTGGAGTTGGTTACTATGTAGAAGGTTTTGAATGTGTCCGTTTTAGTGTCCATTTCTGTGATTTATGCTTCGTTTTGTTCTTGCGAATTTGGTGATCTTGGAATATTCCGGATATGTTGTGTTGATTGTTTGAAACTTTAGCCATTCGTGTGATCTTTATTATCTTGGAACTGATTAAAATTTTAGGGCTTCACTTACAATTGATTCTAGTATTCAGTTTCTGGTTTCTGTTTTGATTCGAAGTTTTTGATTTTTGTAGATGGGATGAGGTGCTTGCGTCTGTTGCTGAGACGATTAAGAACTTTGCAGTCATTTATCTGGGTGGACATCACTGAGGTTCCAGACTTCAACACCATGTACGAGCTGTACGATCCTTCTACGGTCATGTTCTTCTTCAGGAACAAGCACATCATGATCGATCTTGGAACTGGTAACAACAACAAGATCAACTGGGCTCTCAAGGACAAGCAGGAGTTCATTGATATCATTGAGACTGTCTACCGTGGTGCAAGGAAAGGGTCGTGGGTTGGTGATTGCTCCAAAAGATTACTCCACCAAATACCGTTACTAATCGAGCTTCCCAACACTATCTAGTTTGTTAAAACCATTGAGTCTAGTGATTCTGGGTCAGCTGAAATATCCCGTGAACTGATTTCATTCATATATGCTTGATGATGATGTGATTCTGGATTTTGCTACTGTTTCGATGTCCTCATTATGTATGTACTACTGCTCGAACATGAATCAGTGAAATGAAATTGCAGGCGGTTGGATTATGTCTGAATAATATTAATTAGACGTCGGATTCTCGGAGGCGACGCAATGGTGCGATTCCGTAATGAGTCGATTATCCGGCGAAACCAATGCTAGACGATAGTACGAAATGTC

>AT5G19780_16.6b

GCAGGTGTCAGATATCATTCTTATCGTCATGATCAGTTCGAGCAGTTGTCTGCAGTATCACTGGTTGACAGTGCTTCAACTTTCTAACGGACTGACTGAACAGTTCTAGGCCGTTCCTGTGAATTCAACCTACGGTAACCGACAGAGTCGACTTGTATCTTACCGTCACTTTCATACAGAGCAGTTCATTTCGGAGAGAGATTCTGCTTACACACTGGTGAGGAGGACATACTGTGAGATCCAATTTCGCTGTTCACTGTTAAGGTTTGAATGGTTCCTAGAATGCTTTCTGGTATTGCAGGTGGAAAGAAATTGGGATTCTTAGTTCTGACCGTGTGAGAGAAGCTTGCCGACCACTGTACTGCTTACAAGGGTTTTTGGTTTTCAATGCTGTGGTGGTGGAACTGGTTCTTGAATTGGGTTCTTCGTTGCTAGAAGCGTTGTCTGTAAGATTACGGAAAGAAGTCTTAAGCTTGGTTTACCATATTACCTTCTCCTCAGGTACTTTTTCTTTTGCTAGACACTGAAGTAGAACCTCTACATGACATGTTTCTATAGTGGTCTAATTTGGATGAAACTTGGTTTTAATGCATATTCCTCTGTGATGTCAGGTTTCTACTGCTGTTGTAGAGCCTTACAACAGTGTGCTTTCAACGCATTCCTTCTTGAACATACCGATGTAGCTGTCCTCTTGGATAACGAAGCCATCTATGACATTTGCCGCAGATCCCTAGATATCGAGAGGCCAACCTACACAAACTTGAACAGGTTGATATCACAGATCATTTCATCCTTGACAACATCTTTGAGGTTTGATGGTGCCATCAACGTGGATATCACTGAGTTCCAGACCAATCTTGTCCCATATCCCCGTATCCATTTCATGCTGTCATCTTATGCACCAGTCATCTCAGCCGCCAAGGCTTACCACGAGCAGCTATCAGTCCCTGAGATCACCAATGCCGTGTTTGAGCCAGCGAGCATGATGGCAAAGTGTGACCCAAGGCACGGAAAGTACATGGCATGTTGTTTGATGTACCGAGGAGATGTTGTTCCCAAAGATGTTAATGCTGCCGTTGGCACCATCAAGACAAAGAGGACTGTTCAGTTTGTTGACTGGTATGATCTAGAAATGGATATAGCTGCTAATTTTTCTACCTTTATGTTGTTATTATATAGTAACTCCTTTGGCATGGGTTTTGTTTTGCAGGTGCCCAACTGGGTTCAAATGTGGAATCAACTACCAACCTCCAACAGTTGTTCCAGGTGGTGACCTCGCTGAGTTCAGAGAGCTAAGAACCC
